# Strategies for Semiconductor/Electrocatalyst Coupling toward Solar‐Driven Water Splitting

**DOI:** 10.1002/advs.201902102

**Published:** 2020-02-04

**Authors:** Sitaramanjaneya Mouli Thalluri, Lichen Bai, Cuncai Lv, Zhipeng Huang, Xile Hu, Lifeng Liu

**Affiliations:** ^1^ International Iberian Nanotechnology Laboratory (INL) Avenida Mestre Jose Veiga 4715‐330 Braga Portugal; ^2^ Laboratory of Inorganic Synthesis & Catalysis Ecole Polytechnique Federale de Lausanne EPFL ISIC LSCI, BCH 3305 CH‐1015 Lausanne Switzerland; ^3^ School of Chemical Science & Engineering Tongji University 200092 Shanghai P. R. China; ^4^ College of Physics Science & Technology Hebei University 071002 Baoding Hebei P. R. China

**Keywords:** coupling strategies, electrocatalysts, photoelectrochemical water splitting, semiconductor photoelectrodes

## Abstract

Hydrogen (H_2_) has a significant potential to enable the global energy transition from the current fossil‐dominant system to a clean, sustainable, and low‐carbon energy system. While presently global H_2_ production is predominated by fossil‐fuel feedstocks, for future widespread utilization it is of paramount importance to produce H_2_ in a decarbonized manner. To this end, photoelectrochemical (PEC) water splitting has been proposed to be a highly desirable approach with minimal negative impact on the environment. Both semiconductor light‐absorbers and hydrogen/oxygen evolution reaction (HER/OER) catalysts are essential components of an efficient PEC cell. It is well documented that loading electrocatalysts on semiconductor photoelectrodes plays significant roles in accelerating the HER/OER kinetics, suppressing surface recombination, reducing overpotentials needed to accomplish HER/OER, and extending the operational lifetime of semiconductors. Herein, how electrocatalyst coupling influences the PEC performance of semiconductor photoelectrodes is outlined. The focus is then placed on the major strategies developed so far for semiconductor/electrocatalyst coupling, including a variety of dry processes and wet chemical approaches. This Review provides a comprehensive account of advanced methodologies adopted for semiconductor/electrocatalyst coupling and can serve as a guideline for the design of efficient and stable semiconductor photoelectrodes for use in water splitting.

## Graphical abstract

Coupling semiconductor photoelectrodes with electrocatalysts is a common and necessary practice which may substantially improve the photoelectrochemical (PEC) water‐splitting performance. Herein, how the coupling of electrocatalysts to semiconductor influences PEC water splitting is outlined and the strategies developed and adopted so far for semiconductor/electrocatalysts coupling are comprehensively summarized.

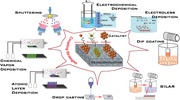

## 1 Introduction

With the ever‐growing world population and their demand for energy, there is a pressing need for a transition from the present fossil‐dominated energy system to a sustainable and carbon‐neutral energy system, in order to avoid the possible catastrophic consequences of global climate change associated with the consumption of fossil fuels.^1^, ^2^ Hydrogen (H_2_) and fuel cell technologies have shown significant potential to empower this transition, able to substantially reduce carbon dioxide (CO_2_) emissions and create a huge new market, as predicted recently by the Hydrogen Council.^3^ Although H_2_ is a clean energy carrier and shows flexibility of linking different energy sectors and energy transmission and distribution networks, the production of H_2_ is currently not that “clean”—nearly 96% of global H_2_ production is from thermochemical processes,^4^ which not only deplete fossil fuels but also emit a large amount of CO_2_. To decarbonize H_2_ production, fossil‐free processes such as electrochemical water splitting using “green” electricity or direct photoelectrochemical (PEC) water splitting must be widely deployed.

PEC water splitting takes advantage of semiconductors as both the light absorber and energy converter, to store solar energy in the form of H_2_ fuel. To make full use of the photogenerated electron–hole pairs, effective separation of electrons from holes and rapid charge transfer from the space charge region to the semiconductor/liquid junction that enables the chemical reaction are very important. For most semiconductors, even if their conduction/valence band edge is properly positioned with respect to the proton reduction potential or the water oxidation potential, the hydrogen evolution reaction (HER) or the oxygen evolution reaction (OER) kinetics on the bare semiconductor surfaces is usually so sluggish that the reaction efficiency is fairly low for practical applications, unless a large overpotential is applied to drive the kinetically rate‐limiting step of the multi‐step reduction/oxidation reaction. In order to enhance the solar‐to‐hydrogen (STH) conversion efficiency of semiconductor photoelectrodes, some strategies such as micro‐ and nanostructuring,^5^ band gap engineering via doping,^6^ usage of co‐catalysts,^7^ and heterostructure construction,^8^ have been investigated, among which coupling semiconductor photoelectrodes with good HER or OER co‐catalysts is a common, and often necessary, strategy to expedite the reaction kinetics and to reduce reaction overpotentials. The addition of catalysts may substantially suppress surface recombination of electrons and holes and/or diminish the accumulation of electrons or holes at semiconductor surfaces that could lead to self‐reduction or self‐oxidation of compound semiconductors. Moreover, some electrocatalysts are also reported to be able to passivate the surface of semiconductors against corrosion in aqueous electrolyte, thereby improving the stability and lifetime of photoelectrodes under operational PEC conditions. Notwithstanding many advantages, the introduction of electrocatalysts may also cause unwanted parasitic light absorption or reflection and fundamentally change the interfacial energetics of semiconductor/liquid junctions, which need to be taken into account when designing a PEC cell.

How to select appropriate semiconductor light absorbers and HER/OER electrocatalysts that can be used to couple with semiconductors for PEC water splitting have been the subject of several recent review articles, and therefore are not covered here.^9^, ^10^ For readers who are interested in the underlying physics of semiconductor/liquid junctions and an overview about the PEC cells, some introductory books and reviews are also available.^2^, ^11^ Given the critical importance of electrocatalysts in PEC water splitting and the fact that loading electrocatalysts on semiconductor photoelectrodes for PEC water splitting has become a common practice, the focus of this review will be placed on summarizing various strategies developed and adopted by far for semiconductor/electrocatalyst coupling. Coupling co‐catalysts to semiconductor powders for photocatalytic water splitting in suspension systems is not covered in this review. Interested readers may refer to previous review articles on this topic.^12^ Before going into details, we will first briefly outline how the catalyst coupling would influence the PEC performance of semiconductors.

## 2 Semiconductor/Electrocatalyst Interface

In general, photocurrent density (*J*_ph_) generated by a semiconductor photoelectrode correlates heavily with illumination intensity (φ), light harvesting (LH) efficiency (η_LH_), charge separation (CS) efficiency (η_CS_), and the efficiency of charge transfer (CT) from semiconductor to electrolyte (η_CT_)

(1) $$J_{\text{ph}}=\varphi \eta_{\text{LH}}\eta_{\text{CS}}\eta_{\text{CT}}$$

**Scheme 1:**
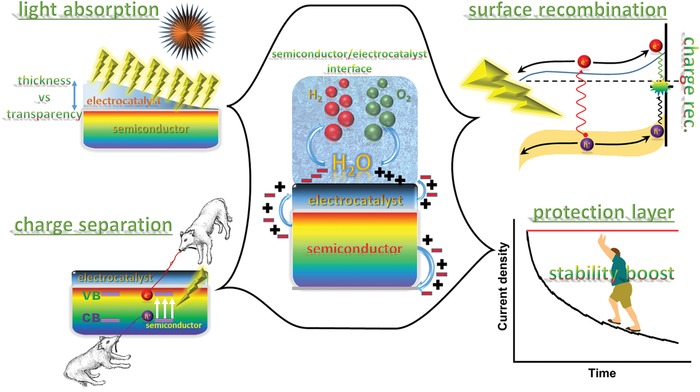
Possible influences of coupling electrocatalysts to a semiconductor photoelectrode.

Since many semiconductors have intrinsically low kinetics for HER or OER, coupling semiconductor light absorbers with suitable electrocatalysts has been proposed to be an effective strategy for promoting η_CT_ and thereby the overall PEC performance of photoelectrodes. Indeed, most catalyst‐modified photoelectrodes have shown an increase in photocurrent density at a given potential, and a marked anodic shift in onset potential for HER or a notable cathodic shift for OER, compared to the bare semiconductor without modification of a catalyst. The coupling of electrocatalysts to semiconductor surface would dramatically change the PEC properties of photoelectrodes (**Scheme** **1**). Depending on the nature of electrocatalysts, the contact between semiconductor and catalyst may form either a Schottky junction (e.g., for metallic catalysts), a semiconductor–semiconductor junction (e.g., for compound catalysts), or an adaptive junction (e.g., for ion‐permeable catalysts).^13^ If the catalysts deposited are discontinuous and have a low coverage and very small feature size, they might be effectively optically transparent and not significantly affect the light absorption of the semiconductor. Otherwise, the influence of catalysts on the PEC performance cannot be ignored.^14^ The catalyst layers or nanoparticles (NPs) may cause parasitic light absorption, blocking a certain portion of incident light to be absorbed by semiconductors. In addition, the catalysts might contribute to the charge separation in the bulk of semiconductors and/or to charge recombination (CR) on the semiconductor surface, besides the charge transfer. In some specific cases, the electrocatalyst contributes even only to the charge separation or recombination, and does not catalyze the redox reactions.^15^, ^16^, ^17^, ^18^ In the following, we will elaborate possible influence of the catalyst addition to semiconductor photoelectrodes on the PEC performance.

### 2.1 Light Absorption

To efficiently convert photogenerated carriers to H_2_ or O_2_ molecules, surface charge transfer efficiency η_CT_ should be improved, which necessitates the increase of catalyst loading. However, most electrocatalysts can absorb/reflect light, and the parasitic light absorption/reflection by catalysts would accordingly reduce η_LH_. It is therefore of importance to decouple light harvesting and charge transfer to simultaneously obtain large η_LH_ and η_CT_.

Modification of semiconductor light absorbers with optically transparent or semitransparent catalysts, e.g., ultrathin layers^19^, ^20^ and wide band gap semiconductors,^21^ is an effective approach to mitigating parasitic light absorption by catalysts. For example, Hu and co‐workers reported that a thin amorphous iron nickel oxide (FeNiO*_x_*) layer can significantly lower the water oxidation onset potential of both thin film and nanostructured hematite (α‐Fe_2_O_3_) photoanodes, but in the meantime does not cause dramatic light absorption because it is optically transparent.^19^ The outstanding optical transparency of the FeNiO*_x_* catalyst layer enabled the preparation of a hematite/perovskite tandem PEC cell, which showed an STH efficiency exceeding 1.9% and a Faradaic efficiency of ≈100% for unassisted water splitting. Jin's group found that MoQ*_x_*Cl*_y_* (Q = S, Se) is a highly active electrocatalyst for HER and a wide band gap semiconductor having very low light absorption in the visible and near‐infrared (NIR) wavelength range. When depositing this semitransparent catalyst on the surface of Si micropyramids (Si‐µP), the resultant photocathode exhibited a record high photocurrent density of 43 mA cm^−2^ at 0 V versus RHE (reversible hydrogen electrode, V_RHE_) among all Si‐based photocathodes reported previously.^21^

**Figure 1:**
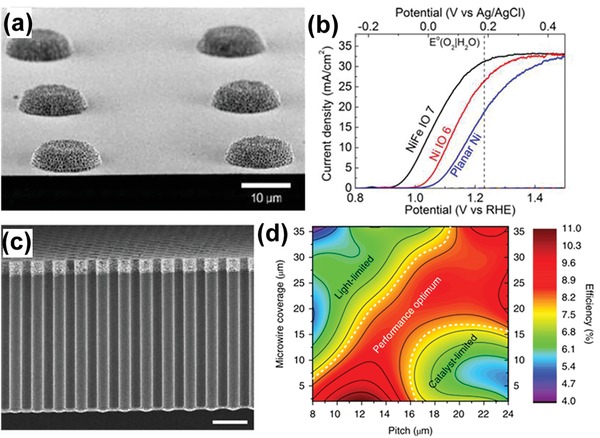
a) Typical SEM image of Si photoanodes loaded with 3D Ni IO catalysts. b) PEC *j*−*V* curves of the oxide‐passivated p^+^ n‐Si photoanode with the micropatterned planar Ni (blue trace), Ni IO (red trace), and NiFe IO (black trace). Reproduced with permission.^22^ Copyright 2017, The American Chemical Society. c) Representative SEM image of Si‐µW arrays with spatioselective coating of Ni‐Mo catalysts (on the top). d) Contour plot of the η_IRC_ as a function of pitch and wire coverage for all tested combinations. Reproduced with permission.^23^ Copyright 2018, Springer Nature Publishing AG.

Another approach to reducing parasitic light absorption of electrocatalysts is to minimize the geometric footprint of the opaque electrocatalysts on the light‐absorbing surfaces of semiconductors.^22^, ^23^, ^24^, ^25^ Constructing three‐dimensional (3D) electrocatalysts on the surface of semiconductor photoelectrodes can relax the constraint on the catalyst loading against catalyst footprint, and therefore decouple the light absorption of semiconductor and surface charge transfer. In this respect, Oh et al. recently presented an example where they loaded isolated islands of Ni inverse opal (IO) on a planar Si photoanode (**Figure** **1**a).^22^ Compared to the planar Ni catalysts having the same coverage on Si, the 3D Ni IO significantly increased the number of catalytically active sites, while not causing extra parasitic light absorption. As a result, a high saturated photocurrent density (*J*_sc_) of 31.2 mA cm^−2^ was achieved and a cathodic shift of photocurrent onset was observed (Figure 1b). In this case, the distances among the catalyst islands need to be optimized to assure the minority carriers to be effectively collected and to avoid excess recombination of the majority carriers. Similarly, Long et al. also demonstrated the benefit of a 3D electrocatalyst structure for the PEC performance of TiO_2_ photoanodes via depositing FeO*_x_* NPs in a matrix of pore‐spanning conducting polymer (CP) on the surface of TiO_2_ nanorod (NR) arrays.^24^ The photocurrent density of the resulting CP‐FeO*_x_*/TiO_2_ was found to improve by a factor of 2.86 relative to that of the pristine TiO_2_ NR array, at 1.23 V_RHE_.

Electrocatalyst footprint reduction can also be accomplished using vertically aligned 3D‐structured semiconductor photoelectrodes. For instance, Lewis and co‐workers loaded Co‐P catalysts on the sidewalls of vertically standing p‐Si microwire (Si‐µW) arrays.^25^ This configuration allows for the minimization of the area of the semiconductor shadowed by the catalyst, thereby reducing parasitic light absorption arising from the catalyst and improving the *J*_sc_. They used the ratio of the *J*_sc_ of p‐Si/Co‐P to that of bare p‐Si to evaluate the parasitic light absorption, and concluded that the parasitic light absorption of Co‐P on p‐Si‐µW surfaces was reduced by a factor of 2 relative to that on planar Si surfaces.^25^ Lately, Huskens et al. developed a method that allows for spatioselectively depositing Ni‐Mo HER catalysts on vertically aligned Si‐µW arrays, which enabled effective decoupling between the catalytic activity and light absorption of photocathode.^23^ They found that the Si‐µW with their upper 2 µm loaded with Ni‐Mo, Figure 1c has PEC performance superior to the Si‐µW with the entire length loaded with Ni‐Mo. Furthermore, based on all samples tested the authors drew a contour showing the solar‐driven HER performance could be optimized by adjusting the µW pitch and the spatioselective Ni‐Mo coverage along the µW (Figure 1d), with the best ideal regenerative cell (η_IRC_) efficiency of 10.8%.

While many electrocatalysts would cause unwanted parasitic light absorption, in some cases the overall light absorption of photoelectrodes may be enhanced if a compound semiconductor electrocatalyst is loaded. For example, Cu_2_S, as an HER catalyst, was deposited on the surface of Cu_2_O nanowire (NW) photocathodes. Comprehensive optical characterization and PEC tests indicated that it also acted as a photosensitizer, extending the optical absorption range of Cu_2_O.^26^

### 2.2 Charge Separation

When photogenerated electrons (e) and holes (h) are formed in semiconductors, they should be effectively separated to avoid recombination before the charge transfer. Electrocatalyst can affect the charge separation process via increasing band bending in the depletion layer of semiconductors,^15^, ^16^, ^27^ forming a p‐n junction^28^, ^29^, ^30^, ^31^ or a metal‐insulator‐semiconductor (MIS) junction^32^ at the semiconductor/electrocatalyst interface.

**Figure 2:**
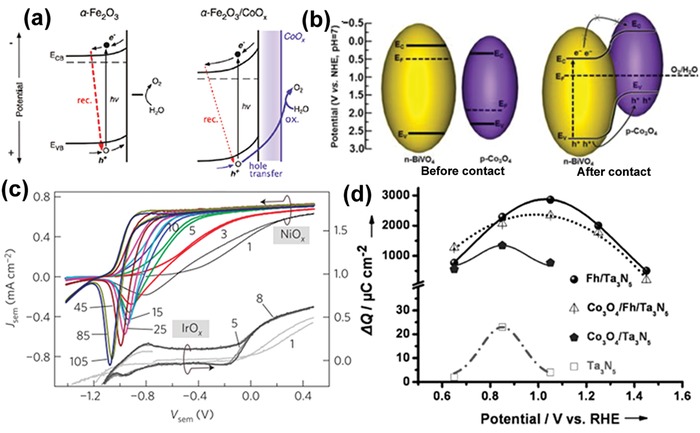
a) Band diagrams illustrating the main fates of charge carriers photogenerated holes in bare α‐Fe_2_O_3_ and α‐Fe_2_O_3_/CoO*_x_*, following light excitation. Reproduced with permission.^27^ Copyright 2011, The American Chemical Society. b) Band diagrams illustrating the charge separation in p‐Co_3_O_4_/n‐BiVO_4_ heterojunction. Reproduced with permission.^28^ Copyright 2015, The American Chemical Society. c) Evolution of the illuminated *J*–*V* curves upon repetitive cycling of TiO_2_/NiO*_x_* and TiO_2_/IrO*_x_* photoelectrodes. Reproduced with permission.^13^ Copyright 2013, Springer Nature Publishing AG. d) Charge storage versus potential curves of Ta_3_N_5_, Fh/Ta_3_N_5_, Co_3_O_4_/Ta_3_N_5_, and Co_3_O_4_/Fh/Ta_3_N_5_ photoanodes. Reproduced with permission.^33^ Copyright 2014, Wiley‐VCH.

Enhanced electron depletion in n‐type semiconductors induced by loading an electrocatalyst can reduce e/h recombination and effectively separate electrons from holes. Barroso et al. found that Co‐Pi can delay the e/h recombination in α‐Fe_2_O_3_, with a transient absorption lifetime (*t*_1/2_) of 30 ms for α‐Fe_2_O_3_/Co‐Pi, in sharp contrast to *t*_1/2_ of 15 µs for bare α‐Fe_2_O_3_.^27^ The dramatic retardation of e/h recombination was attributed to the increased band bending in the α‐Fe_2_O_3_ because of the Co‐Pi‐induced depletion of electrons in the Fe_2_O_3_ conduction band (**Figure** **2**a). The same group investigated the role of CoO*_x_* on the Fe_2_O_3_ photoanode, and revealed that CoO*_x_* did not change the lifetime, but only the yield of long‐lived holes, which is associated with the surface water oxidation.^15^ Accordingly, the performance enhancement in Fe_2_O_3_/CoO*_x_* would not result primarily from the catalytic activity of CoO*_x_*, and instead this enhancement should be attributed to the increased electron depletion of the CoO*_x_* film which mitigates e/h recombination. Durrant et al. further observed the retardation of e/h recombination in BiVO_4_/Co‐Pi.^16^ According to the transient absorption spectroscopy and kinetics analyses, they proposed that there was no hole transfer from BiVO_4_ to Co‐Pi, and therefore no water oxidation occurred via Co‐Pi.

Charge separation can be promoted by the formation of a p‐n junction between the semiconductor and the electrocatalyst. Gong et al. reported the deposition of discontinuous p‐type semiconducting catalyst, namely, Co_3_O_4_, on the surface of n‐BiVO_4_.^28^ The formed p‐n junction increased the charge separation effect in the bulk (Figure 2b), and also overcame the negative effect of Co_3_O_4_, suppressing the formation of recombination centers at the BiVO_4_/Co_3_O_4_ interface. The optimal loading of Co_3_O_4_ enabled a charge separation efficiency of 77% in the bulk and 47% on the surface of catalysts. Similar effect was reported as well in other different couples of electrocatalysts and semiconductors. For instance, Wang et al. showed that BiVO_4_/NiCoO_2_ had a high charge separation efficiency of 72%, much larger than 23% for pure BiVO_4_.^29^ Lee et al. demonstrated that the Ni‐rich NiO*_x_*, as an n‐type semiconductor, was more efficient in catalyzing the HER on the surface of p‐Si in comparison to O‐rich NiO*_x_* and metal Ni film. The formation of p‐Si/n‐NiO*_x_*(Ni‐rich) junction enabled easy charge transfer from Si to NiO*_x_* for the HER.^30^ Sarkar and Singh reported that the axial junction of p‐NiO*_x_*/n‐Fe_2_O_3_ significantly enhanced the photocurrent density and resulted in cathodic shift of the onset potential of the photocurrent.^31^ Due to the formation of a p‐n junction and the existence of NiO*_x_* as an OER catalyst, the time constant of the transient photocurrent of NiO*_x_*/Fe_2_O_3_ was five times longer than that of bare Fe_2_O_3_, suggesting the CR was remarkably suppressed at electrode/electrolyte interface by NiO*_x_*.

Besides p‐n junctions, the MIS junction formed between semiconductor and electrocatalyst also turns out to be able to contribute to efficient charge separation. For example, Smith et al. reported an MIS photoanode consisting of n‐Si/SiO*_x_*/Al_2_O_3_/Pt/Ni, and found that the photovoltage was increased from 385 mV for the fresh sample to 490 mV for the 18 h aged sample,^32^ where the photovoltage was estimated according to the difference between the onset potential of the illuminated photoanode and that of a nonphotoactive electrode (p^+^Si/SiO*_x_*/Pt/Ni) with p^+^Si acting as a conductive current collector. The aging transformed surface metallic Ni to Ni/NiO*_x_*/Ni(OH)_2_, and promoted the surface catalytic activity for the OER. By analyzing the flat band potential (*E*_fb_) derived from Mott–Schottky plot, they found that the calculated barrier height of fresh photoanode is 0.77 eV and that of aged photoanode is 0.9 eV. Hence, the authors attributed the larger photovoltage of aged photoanode to its greater barrier height and corresponding larger surface band bending, which facilitate charge separation. They further rationalized that the oxidized NiO*_x_* on Pt in the aged sample was separated from the insulating layer of Al_2_O_3_, and was therefore not influenced by the pinning effect of Al_2_O_3_. Consequently, the high work function of NiO*_x_*/Ni(OH)_2_ (5.2–5.6 eV) could render a larger barrier height.

In addition to p‐n and MIS junctions, an adaptive semiconductor/electrocatalyst junction, induced by the chemical state changes of ion‐permeable electrocatalysts, may result in adjustable Schottky barrier heights, thereby changing the interfacial energetics. An ion‐permeable electrocatalyst (e.g., nickel oxide)^34^ refers to a catalyst subjected to oxidization to a high valence state during the OER, which may show a different coupling effect on solar‐driven photoelectrocatalysis compared to an ion‐impermeable electrocatalyst (e.g., IrO*_x_*).^13^ Lin and Boettcher first discovered the adaptive behaviors of ion‐permeable NiO*_x_* catalysts when coupled with a TiO_2_ photoanode.^13^ They found that an activation process occurred in the TiO_2_/NiO*_x_* electrode during repetitive cyclic voltammetry (CV) scans, while the CV curves remained unchanged under similar treatment for the TiO_2_ electrode coupled with dense ion‐impermeable IrO*_x_* catalysts (Figure 2c).^13^ During operation, NiO*_x_* was charged through bulk redox reactions (e.g., the oxidation of Ni^2+^ to Ni^3+^), and therefore the hole accumulation resulted in a lower Fermi level in the NiO*_x_* (*E*_cat_) layer relative to both the Fermi level of TiO_2_ (*E*_SEM_) and the energy level of the O_2_/OH^−^ redox couple. Because the barrier height (ϕ_b_) at solid/electrolyte interface was defined as *q* ϕ_b_ = *E*_cat_  − *E*_c_, where *E*_c_ is the conduction band energy of the n‐type semiconductor, a lower *E*_cat_ would lead to a larger ϕ_b_ and thereby an increase in the photovoltage (*V*_OC_) at the TiO_2_/NiO*_x_* interface. In contrast, the effective ϕ_b_ of TiO_2_/IrO*_x_* did not alter because no bulk redox reaction and associated Fermi level moving occurred in this case. As a result, TiO_2_/NiO*_x_* showed PEC performance superior to that of TiO_2_/IrO*_x_*. The researchers further pointed out that the PEC performance of the semiconductor photoelectrodes loaded with hydrous ion‐permeable electrocatalysts is generally expected to show higher PEC performance than that of semiconductors coupled with dense ion‐impermeable catalysts where the buried interface is unlikely to be optimized for charge separation.^13^

### 2.3 Surface Recombination and Fermi Level Pinning

Deposition of electrocatalysts on the surface of a semiconductor can passivate the surface states, and therefore reduce the e/h recombination on semiconductor surfaces. In this respect, Wang and co‐workers reported the deposition of an amorphous NiFeO*_x_* catalyst layer on hematite photoanodes, which resulted in a photovoltage as high as 0.62 V.^35^ The NiFeO*_x_* minimized the surface Fermi level pinning, which prevails in the bare hematite that leads to a low photovoltage. The authors used the intensity‐modulated photocurrent spectroscopy to extract the rate constants of charge transfer (*k*_tran_) and recombination (*k*_rec_), and showed that *k*_tran_ of all hematite electrodes decorated by NiFeO*_x_* is equal to or smaller than that of the bare hematite electrode, indicating that the NiFeO*_x_* catalyst actually did not promote the kinetics of the surface OER.^17^ A significant reduction of *k*_rec_ in NiFeO*_x_*‐modified hematite photoanodes suggests that the function of NiFeO*_x_* is to offset the surface Fermi pinning effect and to passivate the hematite surface against recombination. Similar phenomenon was reported as well by Abdi et al. in BiVO_4_/Co‐Pi photoanodes, in which the role of Co‐Pi was to passivate the surface of BiVO_4_, but not to promote charge transfer (surface OER).^18^

Electrocatalysts sometimes work as a hole storage layer on the surface of semiconductors. For instance, although Fe‐based oxyhydroxide was widely reported to be active for OER, Li et al. found that when loading ferrihydrite (Fh) on the Ta_3_N_5_ photoanode, the charge storage of Ta_3_N_5_ was remarkably increased by at least two orders of magnitude (Figure 2d).^33^ Yang et al. used transient surface photovoltage spectroscopy to investigate the charge transfer/recombination kinetics at the interface of Fe‐NiO nanosheets (NSs) and Mo‐doped BiVO_4_ (Mo‐BiVO_4_), and they found that the ultrathin Fe‐NiO NSs did not improve charge separation rate and suppress the recombination of e/h.^36^ Instead, the p‐type Fe‐NiO greatly increased the maximum accumulated charge on the BiVO_4_ surface, serving as a hole reservoir due possibly to the Ni^2+^/Ni^3 +^ redox pair in the surface of Fe‐NiO. On the other hand, ultrathin Fe‐NiO NSs rendered a higher charge transfer efficiency, resulting in enhanced PEC performance.

It is worth nothing that not all electrocatalysts can help suppress surface recombination and diminish Fermi level pinning. For example, despite the proven electrocatalytic activity of MnO*_x_* for OER, Wang et al. found when depositing MnO*_x_* on the surface of hematite, a significant anodic shift of onset photocurrent density appeared, indicating deteriorated PEC performance.^37^ In this case, MnO*_x_* brings a high density of surface states leading to the surface Fermi level pinning, which reduces the photovoltage of the photoelectrode.

### 2.4 Surface Protection

Since most semiconductor light absorbers, particularly those with narrow band gaps, are not electrochemically stable in aqueous solutions under PEC operational conditions, an additional protection layer is often needed for long‐term H_2_/O_2_ evolution. Chorkendorff and co‐workers have recently reviewed the strategies for protecting semiconductor photoelectrodes.^38^ Interestingly, some electrocatalysts having excellent electrochemical stability in aqueous electrolyte (e.g., noble metals and compound catalysts) that are deposited continuously on the semiconductor surface, can serve as a protection layer preventing the underneath semiconductor from corrosion, thus endowing dual functionality. The duration of stable operation of photoelectrodes relies heavily on the pH of electrolyte as well as the thickness of the catalyst layer. According to Dai group's report, a 2 nm thick catalytic/protective layer of Ni could surprisingly enable continuous PEC water oxidation of n‐Si in weak alkaline solution (pH = 9.5) for 80 h without a decay.^20^ Impressively, Lewis's group demonstrated that deposition of a thick NiO*_x_* multifunctional coating on n‐Si allowed the photoanode to continuously oxidize water under illumination at a large current density of 30 mA cm^−2^ for 1700 h, even in the harsh 1.0 m KOH solution.^39^ Some emerging electrocatalysts, including metal phosphides^40^ and selenides,^41^ have recently shown HER/OER stability of more than 3000 h in alkaline media, which would enable long‐term alkaline PEC water splitting when coupled with semiconductor photoelectrodes; while in acidic electrolyte an additional protection layer is usually still needed for long‐term operation.^42^

## 3 Strategies for Semiconductor/Electrocatalyst Coupling

As discussed above, adding electrocatalysts to semiconductor photoelectrodes may enhance charge transfer kinetics at the semiconductor/liquid junction, suppress surface CR, increase band bending, and in some cases protect semiconductors from corrosion, thereby contributing to the improvement of overall PEC water‐splitting performance. For these reasons, depositing electrocatalysts on photoelectrodes has become a common practice to achieve enhanced PEC performance. To this end, many strategies have been developed by far enabling effective and robust coupling of different HER or OER catalysts to various semiconductor photoelectrodes. These methodologies can be roughly categorized as dry processes and wet chemical approaches, respectively, which will be described in detail in the following.

### 3.1 Dry Processes

Dry processes, including physical vapor deposition (PVD), chemical vapor deposition (CVD), and atomic layer deposition (ALD), have long been used for thin‐film deposition.^43^ Depending on the surface chemistry, roughness, and lattice structures, the deposition may give rise to either a continuous thin film or discontinuous nanometer‐sized islands on the substrate of interest. Dry processes are usually solvent‐free, and therefore enable the deposition of catalysts with clean surfaces. Moreover, most dry processes have been broadly utilized in semiconductor industry and allow for large‐scale fabrication. However, some dry processes (e.g., magnetron sputtering) involve the bombardment of high‐energy atoms or particles on the semiconductor surface, which may cause surface defects or intermixing of catalyst and semiconductor leading to an undesirable semiconductor/catalyst interface. In addition, some dry deposition processes need to be carried out at high temperatures under which the semiconductor's properties may change, which could therefore influence its light harvesting and charge separation capability.

Utilizing dry deposition techniques for semiconductor/catalyst coupling can date back to as early as 1975, at the time Nakato et al. investigated the PEC performance of GaP and Si coated with a number of noble metals including Au, Pd, and Ag, and they observed a gain in onset potential and improved stability for catalyst‐coupled semiconductors compared to the bare ones.^44^ In fact, PVD was the prevailing method used in the early studies of PEC water splitting. It is typically suitable for the deposition of electrocatalysts on a flat, nontextured semiconductor.

#### 3.1.1 PVD

PVD is characterized by a vacuum‐based process in which the material goes from a condensed phase (e.g., a target or an ingot) to a vapor phase and then back to a condensed phase in the form of thin films or islands. The most commonly used PVD processes include evaporation and sputtering. Evaporation involves two basic processes: the source materials first vaporize thermally, and then the evaporated atoms can travel directly to the substrate without colliding with the background gas given their long mean free path in high vacuum. If reactive foreign atoms (e.g., oxygen) are present, the evaporated atoms may react with them leading to the deposition of a compound (e.g., oxide) on the substrate. Compared to evaporation, sputtering involves ejecting materials from a target onto a substrate, by different means such as magnetrons, ion beams, or a gas flow. Sputtering sources contain no hot parts and are compatible with reactive gases allowing for the deposition of complex compounds. Moreover, even materials with very high melting points, which are difficult to evaporate, can be easily sputtered. The sputtered atoms ejected from the target can fly ballistically in straight lines and impact energetically on the substrate. Therefore, typically the sputtered films or NPs have a better adhesion on the substrate than those by evaporation where evaporated atoms are generally soft‐landing on the substrate. Usually, for a substrate with an atomically flat surface, evaporation and sputter may offer unparalleled control over the thickness of the deposited films down to a monolayer. However, PVD is not suited for deposition over large aspect‐ratio substrates or textured substrates with complex 3D configurations, as a high degree of coverage cannot be achieved due to the shadowing effect.

PVD had been widely used to coat platinum group metal (PGM) catalysts on semiconductor photoelectrodes in the early studies of regenerative PEC cells. Typically, Pt, as the best HER catalyst, has been extensively investigated. For example, thin Pt layers were evaporated or sputtered on Si wafers to form a Schottky barrier or MIS junction to increase the open circuit potential and to serve as a protection layer of the semiconductor.^45^ Maier et al. deposited Pt catalyst layers on p‐Si using both electron beam evaporation and magnetron sputtering, and they studied the influence of Pt layers on the PEC performance for HER and compared it to that of p‐Si coated with electrodeposited Pt.^46^ They found that the electron beam evaporated Pt consisted of a homogenous film of pure metallic Pt, while the sputtering resulted in the formation of completely intermixed PtSi*_x_* layer on top of p‐Si although the power density during sputtering was relatively low. Compared to the naked p‐Si, p‐Si photocathodes coated with both evaporated and sputtered Pt showed obvious positive shift in the HER onset potential, confirming the catalytic effect of Pt. Nevertheless, the ultrathin Pt film‐coated p‐Si prepared by PVD did not exhibit HER performance as good as Pt island‐coated p‐Si prepared by photoassisted electrodeposition, due to the formation of ohmic contacts or low Schottky barrier heights in the former, as explained by the researchers.^46^ Similar phenomenon was also observed by Lewis et al. for the planar P^+^‐Si and Si‐µW arrays^47^ as well as by Oh and co‐workers for GaAs photocathodes in their recent work.^48^ In both cases, the e‐beam‐evaporated Pt tends to form an ohmic contact with the underneath semiconductor, resulting in a low or even no photovoltage. Moreover, the optical reflection of the continuous or quasi‐continuous Pt layer attenuates light absorption by the semiconductor, though it can effectively isolate semiconductor from electrolyte improving the photoelectrode's stability. It is worth noting that in addition to the excellent electrochemical stability of its own, Pt catalyst layers can improve the stability of compound semiconductors by reducing the accumulation of photogenerated electrons or holes, which can significantly mitigate photo‐reduction or photo‐oxidation of semiconductors.^49^ While direct deposition of Pt catalysts on semiconductors may change the energetic band alignment, Kaiser et al. managed to sputter Pt catalysts on the ZnO/Ag termination layer of their multi‐junction photoelectrodes.^50^ In this case, Pt couples to the Ag metal surface by simple alignment of the Fermi level without affecting the band structure of Si tandem cells underneath.

Although PVD in general only works well for the deposition of catalysts on flat semiconductor photoelectrodes, attempts were also made to deposit catalysts on high‐aspect‐ratio light absorbers. For example, Pt NPs had been deposited on anodized TiO_2_ nanotube (NT) arrays using DC magnetron sputtering,^51^, ^52^ which demonstrated orders of magnitude improvement in PEC H_2_ evolution rate, compared to bare TiO_2_ NT arrays. Pt loading was found to affect the PEC performance, and a longer deposition time resulted in a decrease in H_2_ production rate which was ascribed to the blocking of photoactive TiO_2_ surface.^52^ It is noteworthy that although the deposition of Pt on the NT's top mouth seemed uniform, no evidence was provided verifying that the deposition happened across the entire thickness of the NT arrays.

In addition to the Pt HER catalysts, PVD was also employed to deposit OER electrocatalysts such as Ir/IrO_2_,^53^, ^54^, ^55^ Ni,^20^, ^56^ and NiO,^39^ on semiconductor photoanodes. For example, Vesborg and co‐workers reported the sputter deposition of Ir/IrO*_x_* thin films directly on p^+^‐n‐Si photoanodes without any other interfacial protective layer.^54^ They found that IrO*_x_* films with a thickness of above 4 nm were highly active for water oxidation in acid electrolyte, but metastable due to an amorphous character. In contrast, a 2 nm IrO*_x_* film deposited on p^+^‐n‐Si was stable, enabling a photocurrent density of 1 mA cm^−2^ at 1.05 V_RHE_. Moreover, heat treatment of sputter‐deposited IrO*_x_* may lead to improved PEC performance. Impressively, despite the absence of an additional protective layer and a low pH electrolyte, the IrO*_x_*/Ir/p^+^‐n‐Si photoanodes could operate stably at 1.23 V_RHE_ under 38.6 mW cm^−2^ simulated sunlight for at least 18 h, highlighting the outstanding dual catalytic/protective role of sputtered IrO*_x_*/Ir thin films. Inspired by this work, Nilsson and co‐workers recently further developed a Si‐based photoanode where a sputtered Au layer was used to interface between p^+^‐n‐Si and sputter‐deposited IrO*_x_*.^57^ The IrO*_x_*/Au/p^+^‐n‐Si showed a lower onset potential, a higher photocurrent, and a higher photovoltage when compared to the previously reported IrO*_x_*/Ir/p^+^‐n‐Si, which the authors ascribed to the rough surface of Au interfacial layer.

Spurgeon et al. sputter‐coated IrO_2_ on WO_3_ photoanodes, and compared their PEC water oxidation performance to the WO_3_ coated with IrO_2_ deposited by other means including spin coating, electrodeposition, and drop casting.^55^ They concluded that sputter coating was the most effective way of coupling IrO_2_ to WO_3_ given that it allowed for a higher degree of surface coverage of catalysts. A thick IrO_2_ layer sputtered on WO_3_ greatly increased the Faradaic O_2_ production efficiency, but meanwhile introduced parasitic light absorption. Moreover, an ohmic contact formed at the IrO_2_/WO_3_ interface, which altered the semiconductor/liquid junction energetics and led to unfavorable photovoltage.

**Figure 3:**
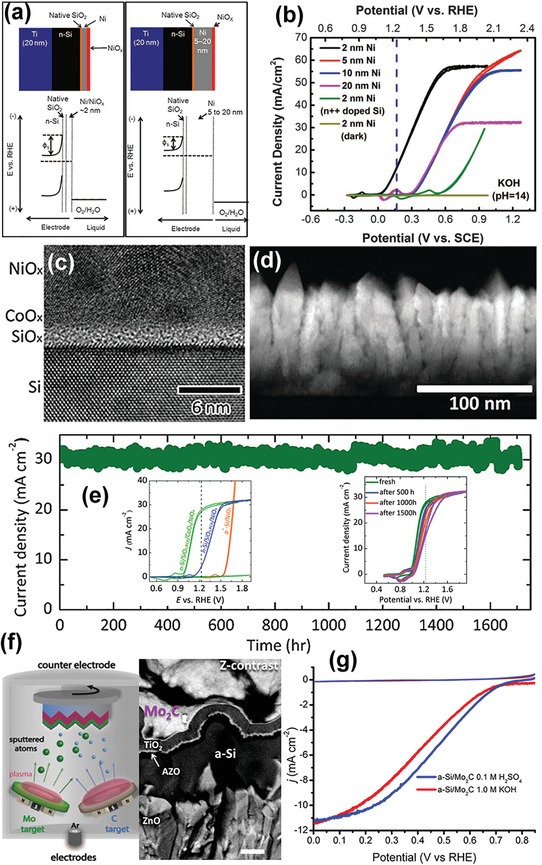
a) Schematic illustration of the band structure of 2 nm Ni‐coated (left) and 5–20 nm Ni‐coated (right) *n*‐Si photoanodes and the corresponding proposed energy band diagram. b) CV curves of 2, 5, 10, and 20 nm Ni‐coated *n*‐Si anodes in 1 m KOH under illumination with a xenon lamp. Reproduced with permission.^20^ Copyright 2013, The American Association for the Advancement of Science. c) High‐resolution transmission electron microscope (HRTEM) image of the cross‐section of *n*‐Si/SiO*_x_*/CoO*_x_*/NiO*_x_* photoanodes. d) Low‐magnification high‐angle annular dark‐field scanning transmission electron microscopy (HAADF‐STEM) cross‐sectional image of the *n*‐Si/SiO*_x_*/CoO*_x_*/NiO*_x_* electrode. The bright film is the polycrystalline NiO*_x_* layer, which grows in a columnar fashion with vertical grain boundaries. e) Chronoamperometry of *n*‐Si/SiO*_x_*/CoO*_x_*/NiO*_x_* photoanodes biased at 1.63 V_RHE_ under 1 Sun of simulated 1.5G solar illumination. Inset: representative *J*–*E* curves of *n*‐Si/SiO*_x_*/CoO*_x_*/NiO*_x_* and *n*‐Si/SiO*_x_*/NiO*_x_* photoanodes measured in 1.0 m KOH(aq) in the dark and under 100 mW cm^−2^ illumination (left); representative *J*–*E* curves of an *n*‐Si/SiO*_x_*/CoO*_x_*/NiO*_x_* photoanode measured before and after 500, 1000, and 1500 h of continuous operation at 1.63 V_RHE_ (right). Reproduced with permission.^39^ Copyright 2015, The Royal Society of Chemistry. f) Schematic illustration of the deposition of Mo_2_C catalysts by confocal magnetron co‐sputtering onto a‐Si photocathodes (left); Cross‐sectional SEM image acquired using an energy selective backscattered detector, showing the configuration of the photocathode: Mo_2_C (5 nm)/TiO_2_ (100 nm)/AZO (20 nm)/a‐Si (300 nm)/ZnO (2 µm). Scale bars: 200 nm. The angle of view is 45°. g) Linear sweep voltammetry (LSV) curves for the a‐Si/Mo_2_C photocathode in 1.0 m KOH and 0.1 m H_2_SO_4_ under simulated AM1.5 (1 Sun) illumination. The sweep rate is 5 mV s^−1^. Reproduced with permission.^58^ Copyright 2015, The American Chemical Society.

While Ir‐based catalysts indeed show high OER activity, their large‐scale usage is prohibitively restricted by the high cost and low natural abundance. For this reason, many attempts have been made toward the development of photoanodes coupled with non‐PGM OER catalysts. Toward this end, Ni‐based catalysts have been recently extensively investigated for PEC water oxidation. In fact, the study of Ni‐modified n‐Si photoanodes can date back in the late 1980s.^56^ In 2013, Dai and co‐workers re‐investigated this strategy and studied how the Ni film thickness and the electrolyte composition influence PEC water oxidation of Ni/n‐Si photoanodes (**Figure** **3**a).^20^ They found that a 2 nm thick Ni film evaporated on n‐Si by e‐beam results in a high photovoltage of ≈300 mV due to the high effective work function of Ni/NiO*_x_*/electrolyte interface (Figure 3b). Moreover, the thin Ni layer evaporated serves as both an efficient OER catalyst and an effective protection layer, enabling the photoanode to operate at 10 mA cm^−2^ in a weak alkaline solution (0.65 m K‐borate and 0.35 m Li‐borate, pH = 9.5) for 80 h without degradation. However, in the highly corrosive 1.0 m KOH electrolyte, the photoanode starts to degrade after 24 h of continuous operation. Researchers found that a thicker Ni film (e.g., 5 nm) may alleviate corrosion at high pH, but suffers from low photovoltage due to the low Schottky barrier height formed by Si/SiO*_x_*/Ni. Smith et al. recently proposed an MIS configuration that allows to decouple the metal properties for Schottky junction formation.^32^ They introduced a high‐quality tunneling Al_2_O_3_ layer capable of substantially reducing the Fermi‐level pinning and allowing for Schottky junction formation between the metal and n‐Si. Moreover, they sputter‐coated a bi‐metal layer of a high work function Pt and a catalytic layer of Ni on Al_2_O_3_ protected n‐Si, in such a way they were able to achieve a Schottky barrier height as high as 0.9 V and realize stable operation at photocurrent densities above 20 mA cm^−2^ in highly corrosive 1.0 m KOH for 200 h.

Motivated by Dai group's work, Lewis's group reported the deposition of nickel oxide (NiO*_x_*) films on a variety of semiconductor surfaces including p^+^n‐Si, hydrogenated amorphous Si (a‐Si:H), CdTe, and p^+^n‐InP using reactive sputter deposition, as both a protective and catalytic layer.^59^, ^60^ NiO*_x_* is optically transparent in the visible region and has a refraction index that makes NiO*_x_* a near‐optimal antireflective coating layer. Furthermore, NiO*_x_* is chemically stable at high pH and upon activation forms a highly OER‐active layer. With the reactively sputtered NiO*_x_* layer, the p^+^n‐Si(100) photoanode was able to show photocurrent‐onset potentials of −180 ± 20 mV relative to 1.23 V_RHE_, photocurrent densities of 29 ± 1.8 mA cm^−2^ at 1.23 V_RHE_, and a solar‐to‐O_2_ conversion figure‐of‐merit of 2.1%.^59^ Impressively, the photoanode operated over 1200 h under light‐driven water oxidation conditions in 1.0 m KOH, showing superior stability. Furthermore, the same group engineered the interfacial energetics between n‐Si and the sputtered NiO*_x_* by introducing a thin, compositionally controlled cobalt oxide layer in between (Figure 3c,d).^39^ The engineered n‐Si even without a buried homojunction can yield open circuit potential values close to the Shockley diode limit and demonstrate 1700 h stability upon continuous operation at 30 ± 2 mA cm^−2^ under illumination conditions (Figure 3e).^39^ Interestingly, e‐beam‐evaporated NiO*_x_* was recently reported to be able to become an electrolyte‐permeable porous layer, thereby forming an adaptive junction with the underlying Si, which can lead to a very large open circuit voltage.^61^

Besides the conventional metal‐ or metal oxide‐based HER and OER catalysts, metal nonoxides have recently drawn considerable attention for use to catalyze the HER and OER, which have demonstrated catalytic performance comparable to that of PGM counterparts.^10^, ^58^, ^62^ For example, molybdenum sulfide (MoS_2_) and selenide (MoSe_2_) were studied intensively for use as an HER or OER catalyst given their decent catalytic activity and outstanding electrochemical stability in a wide pH range,^63^, ^64^, ^65^, ^66^ and therefore many efforts have recently been made to couple semiconductor light absorbers with MoS_2_ or MoSe_2_ using PVD.^65^, ^67^, ^68^ Shen's group reported direct deposition of MoS_2_ and MoSe_2_, respectively, on n^+^p‐Si photocathodes using magnetron sputtering.^65^, ^68^ Upon sputtering, a continuous, vertically standing, grass‐like MoS_2_ or MoSe_2_ layer with abundant exposed active edge sites was formed on Al_2_O_3_ passivated pyramidal n^+^p‐Si wafers. The as‐fabricated MoS_2_/Al_2_O_3_/n^+^p‐Si photocathode could generate a photocurrent density of −32.4 mA cm^−2^ at 0 V_RHE_ with an outstanding catalytic stability of 120 h for the HER under illumination,^68^ due to the formed favorable band structure, which allowed better charge transfer from Si to MoS_2_. Besides direct deposition, MoS_2_ catalyst was also reported to couple with semiconductor photoelectrodes using a two‐step procedure. Chorkendorff and co‐workers first reported this method.[qv: 67b] They sputtered a thin layer of metallic Mo onto n^+^p photocathodes, and then did thermal sulfurization treatment in a gas mixture of H_2_S and H_2_ at 450 °C. Under these conditions, the Mo layer could not be fully converted into MoS_2_, and therefore a MoS_2_/Mo/n^+^p‐Si architecture was formed. The MoS_2_/Mo/n^+^p‐Si showed a saturation photocurrent density of −12 mA cm^−2^ and an HER onset potential of ≈0.25 V_RHE_. Remarkably, the photocathode could operate for 120 h without decrease in photocurrent in strong acidic solution (1 m HClO_4_), exhibiting very good stability. It is interesting to note that in this work no additional protective layer was deposited on n^+^p‐Si, and the MoS_2_ layer offers dual functions as both an electrocatalyst and a passivation layer. Similar approach was also employed later on to couple MoS_2_ with GaInP_2_/GaAs,^69^ which substantially improved the activity and stability of the photocathode.

Some metal nonoxide catalysts, e.g., carbides, are not easy to be conformally loaded on semiconductor photoelectrodes using wet chemical approaches. In this regard, PVD offers a good alternative. Hu and co‐workers recently reported magnetron co‐sputtering of Mo_2_C catalyst layers on amorphous Si (a‐Si) light absorbers (Figure 3f).^58^ Compared to crystalline Si, a‐Si has an optical band gap (Tauc bandgap) of 1.6–1.8 eV, and therefore can provide a high photovoltage of up to 0.93 V for solar‐driven HER. Via co‐sputtering, Mo_2_C formed a continuous and conformal layer on the protected a‐Si (TiO_2_/AZO/a‐Si), and the as‐fabricated photocathode showed outstanding HER performance in both acidic and alkaline electrolytes (Figure 3g). While the co‐sputtered Mo_2_C demonstrated very good long‐term stability in 1.0 m KOH solution, the photocurrent density of Mo_2_C/TiO_2_/AZO/a‐Si photocathode was found to decrease gradually over time, which could be attributed to instability of TiO_2_ and AZO layers in the corrosive alkaline electrolyte. The researchers proposed that a better stability could be achieved by improving the crystallinity of TiO_2_ or introducing a more corrosion‐resistant Mo interlayer.^58^

Transition metal phosphide (TMP) is another emerging class of electrocatalysts that are active for both HER and OER.^70^ While direct PVD of TMP on semiconductors has not been reported, the coupling of TMP with semiconductors is usually achieved via a two‐step procedure, namely, PVD of metallic films and subsequent phosphorization treatment. For example, Li and co‐workers deposited a layer of Ni on the pyramid textured surface of pn^+^‐Si/Ti and then converted Ni into NiP_2_ in a post‐phosphorization treatment using red P vapor.^71^ The as‐fabricated pn^+^‐Si/Ti/NiP_2_ photocathode demonstrated an HER onset potential of 0.41 V_RHE_, a decent photocurrent density of 12 mA cm^−2^ at 0 V_RHE_, and operational durability of 6 h in 0.5 m H_2_SO_4_ solution. Using a similar approach, Jin et al. further fabricated a CoPS‐coated n^+^‐p‐p^+^‐Si photocathode which exhibited an impressive Faradaic STH production efficiency of 4.7%.^72^

#### 3.1.2 CVD

Similar to PVD, CVD is also a vacuum‐based deposition technique that has been broadly used in both semiconductor industry and academic research. In a typical CVD process, the substrate is exposed to one or more volatile precursors, which react and/or decompose on the surface of the substrate to produce desired deposits. CVD can be practiced in a variety of formats depending on the means of initiation of chemical reactions. Compared to PVD, one of the biggest advantages of CVD is that the deposition can happen on irregular surfaces, sometimes even on high‐aspect‐ratio nanostructures. However, nearly all CVD processes take place at a high temperature, so CVD can only be used to couple electrocatalysts to semiconductors in the case the intrinsic properties of semiconductor materials (e.g., light absorption, band structure, and conductivity) are not sensitive to high temperatures and/or the semiconductor photoelectrode is properly protected.

**Figure 4:**
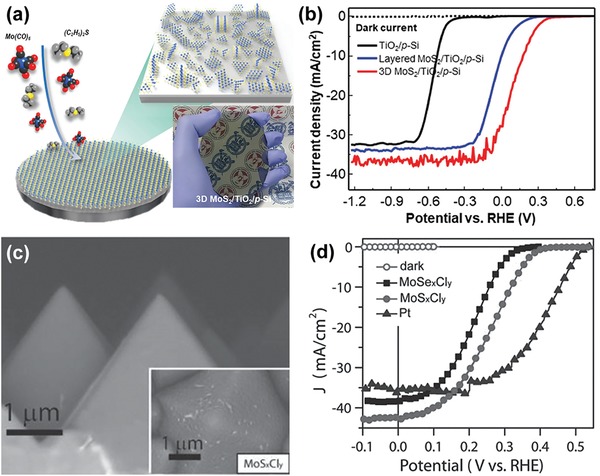
a) Schematic illustration of direct growth of 3D MoS_2_ film on a 4 in. TiO_2_/p‐Si wafer by MOCVD. Inset: Digital photograph showing the 3D MoS_2_ film modified TiO_2_/p‐Si wafer. b) LSV curves of MoS_2_/TiO_2_/p‐Si and TiO_2_/p‐Si photocathodes. Reproduced with permission.^76^ Copyright 2018, Wiley‐VCH. c) Cross‐sectional SEM images of MoS*_x_*Cl*_x_*/Si‐µPs. Inset: top‐view SEM image. d) *J*–*E* curves for MoS*_x_*Cl*_x_*/Si‐µPs (circles), MoSe*_x_*Cl*_y_*/Si‐µPs (squares), and Pt/Si MPs (triangles) photocathodes measured in 0.5 m H_2_SO_4_ under simulated 1 Sun irradiation. Reproduced with permission.^21^ Copyright 2015, Wiley‐VCH.

Given the ease of CVD growth of Mo‐based catalysts and their decent catalytic performance for HER or OER, Mo‐based catalysts including MoS_2_,^73^, ^74^, ^75^, ^76^ MoO_3_,^77^ MoS*_x_*Cl*_y_*,^21^, ^78^ have been explored to couple with semiconductor photoelectrodes for PEC water splitting. Using CVD, Zhang et al. deposited MoS_2_ NSs on Ag@Si nanosphere array photocathodes, using MoO_3_ and sulfur (S) powders as the reaction precursors.^73^ They found that the obtained Ag@Si/MoS_2_ photocathode with optimized MoS_2_ loading exhibited much improved PEC performance for HER, compared to the Ag@Si and MoS_2_‐modified planar Si electrodes. Furthermore, the group reported the fabrication of SiNW photocathodes coupled with MoS_2_ nanoflakes grown by a similar CVD procedure.^74^ Although the basal planes of MoS_2_ were parallel to the NW axis so that the catalytically active edge sites were not preferentially exposed, the as‐synthesized SiNWs/MoS_2_ photocathode still showed reasonably good PEC HER performance as compared to those reported in the literature (without buried junctions), with a photocurrent density of 16.5 mA cm^−2^ achieved at 0 V_RHE_ and an impressive long‐term stability of 48 h in 0.5 m H_2_SO_4_. Yang and co‐workers recently developed a MoS_2_/TiO_2_ heterostructured photoelectrode that shows remarkable PEC performance for light‐driven HER.^75^ The photoelectrode was prepared by PVD of metallic Mo on the anodized TiO_2_ nanocavity arrays, followed by a CVD process converting the Mo into crystalline MoS_2_. Interestingly, in this way the MoS_2_ nanoflakes grew perpendicularly to the wall of TiO_2_ NTs exposing a large portion of catalytically active sites. More importantly, this deposition strategy enables significant enhancement in the absorption of visible light due to the local surface plasmon resonance arising from collective oscillations of excess charges on the edge of MoS_2_. Coupling MoS_2_ to p‐Si using CVD was even demonstrated on a wafer scale.^76^ Using metal‐organic CVD (MOCVD), Andoshe et al. deposited 3D MoS_2_ films with numerous exposed edge sites on a 4 in. TiO_2_‐coated p‐Si wafer (**Figure** **4**a). The CVD‐grown MoS_2_ thin films not only substantially reduced the HER overpotential of Si photocathodes but simultaneously increased the saturation current density due to the anti‐reflection effect. Moreover, MoS_2_ helped stabilize the photocathode under operational conditions in 0.5 m H_2_SO_4_. Consequently, the 3D MoS_2_/TiO_2_/p‐Si photocathode showed a short‐circuit photocurrent density as high as 28 mA cm^−2^ at 0 V_RHE_, an onset potential of 0.35 V_RHE_, and outstanding stability of 181 h continuous operation without noticeable degradation (Figure 4b).

While the CVD growth of MoS_2_ usually happens at a relatively high temperature (e.g., above 500 °C),^73^, ^74^, ^76^ a low‐temperature CVD will be preferable and allow for the integration of catalysts without affecting the intrinsic properties of semiconductor light absorbers. Jin's group recently reported low‐temperature CVD growth of MoQ*_x_*Cl*_y_* (Q = S, Se), an emerging efficient HER catalyst.^21^, ^78^ They used MoCl_5_ and S as precursors and successfully deposited an amorphous layer of MoS*_x_*Cl*_y_* at 275 °C on a planar Si and a micropyramid‐textured Si substrate (Si‐µP) with buried junctions (Figure 4c), respectively. In addition to the high HER performance, the MoS*_x_*Cl*_y_* was also confirmed to be a wide band gap semiconductor, and therefore showed very low parasitic light absorption in the visible and NIR region.^21^ As a result, the MoS*_x_*Cl*_y_*/Si‐µP photocathode demonstrated the highest ever photocurrent density of −43 mA cm^−2^ at 0 V_RHE_ with reasonably good stability of 2 h (Figure 4d).^21^ Moreover, the researchers pointed out that CVD enables the formation of higher quality interfaces with smaller charge transfer resistance, compared to the samples made by drop casting.

Aside from Mo‐based catalysts, metal oxide and recently emerged phosphides were also reported to be able to couple with semiconductor photoelectrodes using CVD. For instance, Jun et al. deposited a thin layer of Ti‐doped α‐Fe_2_O_3_ on n^+^n‐Si through a low‐temperature CVD process using Fe(CO)_5_, Ti[OCH(CH_3_)_2_]_4_, and O_2_ as precursors, which achieved high PEC water oxidation performance showing a maximal photocurrent density of 17.27 mA cm^−2^ at 1.23 V_RHE_ at pH = 13.8. The CVD‐deposited α‐Fe_2_O_3_ was confirmed to serve as an efficient OER catalyst and a 10 nm thickness allowed a significant portion of incident light to penetrate to be absorbed by Si.^79^ Lately, Whitmire and co‐workers employed MOCVD to deposit ternary FeMnP as an OER catalyst on TiO_2_ NRs grown on fluorine‐doped tin oxide (FTO) substrates. A single‐source precursor FeMn(CO)_8_(µ‐PH_2_) was used for the deposition, which can guarantee the homogeneity of the deposited phases given that the stoichiometry is defined in the precursor. FeMnP was found to cover the TiO_2_ NRs along almost the entire length, highlighting the advantage of MOCVD for depositing materials over 3D‐textured substrates. The photocurrent density generated by TiO_2_/FeMnP photoanode reached 1.8 mA cm^−2^ at 1.23 V_RHE_ under 1 Sun illumination, the theoretical maxima of rutile TiO_2_.

While CVD is mostly used to grow compound catalysts on semiconductors, it was reported that elemental catalysts could also be deposited on light absorbers using CVD technique. For example, metallic Ni NPs and thin layers had been deposited on S‐doped TiO_2_ thin films^80^ and TiO_2_ protected n‐Si,^81^ respectively, in both cases improved PEC performance was observed. Recently, carbon NWs (CNWs) were also reported to grow on SiNW photocathodes using CVD.^82^ The obtained CNW‐SiNW hierarchical heterostructures indeed showed improved PEC performance for HER. Although CNWs were claimed to be “co‐catalysts,” no data about the dark electrocatalytic activity of CNWs were provided. Therefore, whether CNWs can really catalyze the HER needs to be further verified.

#### 3.1.3 ALD

ALD is a thin‐film deposition technique based on the sequential use of a gas‐phase chemical process. A typical ALD reaction involves two or more precursors that react with the surface of a substrate one at a time in a sequential, self‐limiting manner. Repeated exposure of substrates to separate precursors, a thin film is slowly deposited. Given the self‐limiting nature, ALD in principle enables a control over the film thickness with a high precision. Moreover, ALD allows for conformal deposition of a pinhole‐free film on a substrate with complicated 3D configuration, and therefore has been widely used for surface and interface engineering of nanostructures in recent years. Dependent on the surface chemistry of substrates and the deposition cycle numbers, ALD may result in either a continuous thin film or discrete NPs. The use of ALD for electrocatalyst synthesis was recently summarized in a book chapter.^83^ These features make ALD a promising technique to deposit various electrocatalysts, e.g., Pt NPs,^84^, ^85^, ^86^ cobalt oxide,^39^, ^87^, ^88^, ^89^ nickel oxide,^90^, ^91^ and molybdenum sulfide,^92^ on the surface of different semiconductors to enhance the PEC water‐splitting performance.

**Figure 5:**
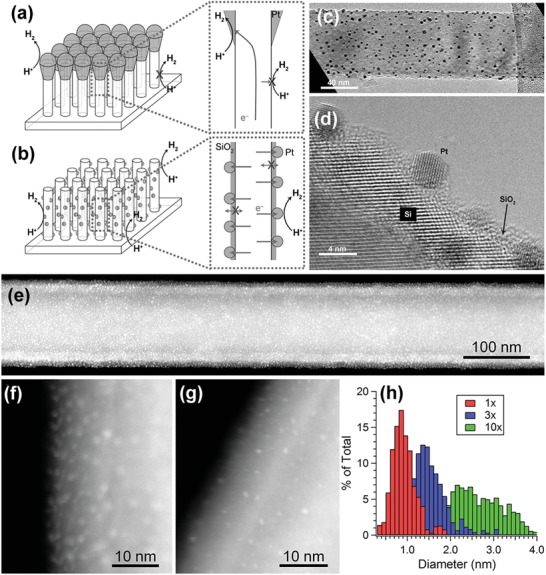
Schematic illustration of the morphology differences between Pt NP catalysts produced a) by ELD and b) by ALD. c) TEM and d) HRTEM images of SiNWs decorated with ALD Pt. Reproduced with permission.^84^ Copyright 2013, Wiley‐VCH. e−g) HAADF‐STEM images of an NW after e) ten cycles, f) three cycles, and g) one cycle of Pt ALD. h) Histogram of particle size for various numbers of ALD cycles. Reproduced with permission.^85^ Copyright 2013, The American Chemical Society.

When depositing NPs on a substrate, ALD usually offers much more uniform dispersion as compared to other wet chemical methods. For this reason, ALD has been widely used to couple nanoparticulate catalysts to different semiconductor photoelectrodes. For example, Wang et al. used ALD to deposit Pt NPs on the surface of SiNW photocathodes, and compared the PEC performance to that of SiNW electrodes coupled with Pt via electroless deposition (ELD).^84^ While the ELD Pt was only found on the tip of SiNWs, ALD enabled uniform deposition of ultrasmall (≈3 nm) Pt NPs on the sidewall of the entire SiNWs (**Figure** **5**a–d).^84^ The uniform distribution of ALD Pt NPs is beneficial for efficient collection of photogenerated charges across the radial direction. As a consequence, the turn on potential (a potential vs RHE to produce a photocurrent density of 1 mA cm^−2^) of SiNWs with ALD Pt was 0.03 V more positive than that of SiNWs with ELD Pt, and the fill factor of SiNWs with ALD Pt was 55%, much larger than that of SiNWs with ELD Pt (28%). However, the saturated photocurrent of SiNWs with ALD Pt was found to be significantly lower than that of SiNWs with ELD Pt (9.0 vs 23 mA cm^−2^). Wang et al. attributed this reduction to the poor diffusion of H^+^ or H_2_ or both through the densely packed SiNWs.

Another distinct feature of ALD is the capability of precise control of catalyst loading on the semiconductor surface. This is of particular importance for reducing the utilization of precious metal catalyst in solar water‐splitting cells. In this respect, Yang and co‐workers reported that using ALD the loading of Pt on SiNWs could be decreased down to 10–100 ng cm^−2^ (Figure 5e–h).^85^ ALD‐deposited Pt was also used to promote the PEC performance of other photocathodes than Si. For example, Geyer et al. deposited Pt on CdSe photocathodes using ALD, which generated a saturated photocurrent density of −1.08 mA cm^−2^ under AM 1.5G illumination, being 12 times that of bare CdSe films.^86^ Meanwhile, ALD of Pt significantly improved the stability of the photocathode. The CdSe film with ALD Pt showed only 8.3% performance degradation in acidic electrolyte in 6 h. In contrast, the CdSe with electrodeposited Pt exhibited 80% performance degradation under similar testing conditions.^86^

ALD enables conformal deposition of a range of transition metal oxides such as CoO*_x_*,^39^, ^87^, ^88^, ^89^, ^93^, ^94^, ^95^ and NiO*_x_*,^90^, ^91^ which can be used as effective catalysts in PEC water splitting. Moreover, the crystal structure and stoichiometry of the deposited oxide layers can be tuned by adjusting the deposition conditions. Notably, beside the role in electrocatalysis, some ALD thin oxide films such as CoO*_x_* may exhibit multiple functions when deposited on semiconductor photoanode surfaces, for instance, forming a heterojunction with semiconductor to produce a high photovoltage, passivating surface states to suppress CR, and/or acting as a protection layer against corrosion.^39^, ^87^, ^88^, ^89^ Sharp et al. reported plasma‐enhanced ALD of a tailored biphasic Co_3_O_4_/Co(OH)_2_ thin film comprising compact and continuous nanocrystalline Co_3_O_4_ spinel and a structurally disordered Co(OH)_2_ layer on its top. When depositing the biphasic film on a p^+^‐n‐Si photoanode, they achieved the highest PEC activity (*J*_sc_: 37.5 mA cm^−2^, photovoltage: 600 mV) reported so far for crystalline Si photoanodes as well as long‐term stability of at least 72 h in 1 m KOH.^88^ The authors found that unlike traditional layered hydroxides, which are susceptible to drying‐induced cracking and delamination, the biphasic Co_3_O_4_/Co(OH)_2_ thin film was simultaneously robust and active, thanks to the unique plasma‐enhanced ALD process taking place at a low temperature. In addition, it was reported that the role of cobalt oxide in PEC water splitting can be engineered by ALD temperature. Oh et al. observed that CoO grown at a low temperature (150 °C) could be used to build a hole‐selective heterojunction with n‐Si, and Co_3_O_4_ formed at a high temperature (300 °C) acted as an efficient catalyst for the OER.^89^ On this basis, they constructed a SiO*_x_*/n‐Si photoanode loaded with a double‐layered ALD CoO*_x_* film (CoO and Co_3_O_4_), which exhibited a *J*_sc_ of 3.5 mA cm^−2^, a value twofold higher than that of the CoO‐modified SiO*_x_*/n‐Si photoanode. Besides Si, ALD‐derived CoO*_x_* films were also reported to couple with other semiconductor photoanodes, e.g., BiVO_4_,^93^ InGa/GaN,^94^ and Fe_2_O_3_,^95^ and enhanced PEC performance was observed in all cases.

Similar to CoO*_x_*, nickel oxide is also often used as an efficient OER catalyst and has been deposited by ALD on different semiconductor photoanodes. For example, Domen et al. deposited NiO on a CoO*_x_*‐modified BiVO_4_ photoanode (CoO*_x_*/BiVO_4_). The conformally coated NiO NPs by ALD were transformed to NiOOH, which is highly active for OER. Moreover, the authors presumed that a small amount of Co was possibly incorporated into NiOOH, leading to a further increase in the electrocatalytic activity. As a result, the fabricated NiO/CoO*_x_*/BiVO_4_ showed a half‐cell solar‐to‐H_2_ energy conversion efficiency of over 1.5% under AM 1.5G illumination.^90^ Clemens and co‐workers decorated GaAs NW arrays with a thin layer of ALD‐deposited NiO*_x_*, and they demonstrated that the NiO*_x_* layer acted as both an OER catalyst and a protection layer of GaAs, which enhanced the PEC water oxidation activity and operational stability of the photoelectrode.^91^

Besides noble metals and metal oxides, metal nonoxide catalysts such as sulfides and nitrides can also be deposited by ALD to improve the PEC performance of photoelectrodes. Oh et al. reported an efficient Si photocathode coupled with ALD‐derived MoS_2_ catalysts for PEC HER.^92^ They found that the size of MoS_2_ NPs and thickness of MoS_2_ thin films could be readily controlled by ALD reaction cycle numbers, and the stoichiometry, crystallinity, and number of catalytically active edge sites of MoS_2_ were able to be tuned by adjusting the post‐sulfurization temperatures.^92^ The optimized MoS_2_/Si photocathode exhibited a saturated photocurrent density of 31 mA cm^−2^, larger than that of the Si photocathode loaded with ELD Pt. Park et al. utilized plasma‐enhanced ALD to deposit a TiN layer of 2–20 nm on the surface of p‐Si‐µWs and demonstrated that the TiN layer had multiple functions, which acted as an electrocatalyst for HER, an antireflection layer, and a protection layer, all contributing to the improved performance of Si‐µW/TiN heterojunction photocathodes.^96^

In some cases, it is difficult to directly deposit a nonoxide catalyst of interest using ALD due to the unavailability of suitable ALD precursors and/or the unfavorable harsh deposition conditions. In this case, the coupling of electrocatalysts to semiconductors can be realized via a two‐step process, namely, ALD of the corresponding oxide on the surface of photoelectrodes, followed by a post‐treatment that converts the oxide into the active catalyst of interest. For example, Narkeviciute and Jaramillo used thermal ALD to deposit a Ta_2_O_5_ layer on SiNWs, and then converted it into Ta_3_N_5_ through nitridation treatment at 800 °C in NH_3_.^97^

### 3.2 Wet Chemical Processes

Wet chemical process is another promising way to deposit catalysts on semiconductor photoelectrodes. In general, coupling semiconductors with electrocatalysts via a wet chemical process involves wet chemical treatment of semiconductors in a certain chemical bath containing metal ions and other auxiliary precursors under desired conditions. In some cases, post‐treatment is needed to ensure good adhesion of catalysts to the semiconductor or achievement of improved catalytic performance. In comparison to the dry processes that usually require expensive equipment and high‐vacuum conditions, wet chemical processes are easily accessible for common chemistry laboratories and have the potential for large‐scale production with low cost. Typically, the most commonly used wet‐chemistry approaches to semiconductor/catalyst coupling include: 1) electrochemical deposition (ECD); 2) ELD; 3) dip coating; 4) successive ionic layer adsorption and reaction (SILAR); 5) hydrothermal and solvothermal treatment; 6) droplet‐based methods (drop casting, spin coating, and spray coating), which will be described in detail below.

#### 3.2.1 ECD

ECD is one of the most widely used wet chemical methods to integrate HER or OER catalysts on photoelectrodes. ECD is simple, fast, cheap, and easily scalable. Typically, the catalytic materials can be coated on photoelectrodes by either voltammetric (CV and linear scan voltammetry—LSV) or galvanostatic/potentiostatic deposition. The catalyst loading is roughly controlled by the passed charges and the Faradaic efficiency of electrodeposition. In general, the semiconductor light absorber needs to be electrochemically stable under the deposition conditions.

Unlike the ECD on conductive substrates where only small overpotentials are required to overcome the nucleation barriers, ECD on semiconductors usually needs a large overpotential to drive, given the comparatively low electrical conductivity and the low surface charge carrier concentration of semiconductors. Typically, the overpotential required for a metal catalyst to deposit on a semiconductor depends on the relative position of the redox potential of the metal with respect to the flat band potential of the semiconductor, and the nucleation behavior of the metal is influenced by the difference in work functions of the depositing metal and the substrate as well as the chemical interaction between them.^98^

Given the above‐mentioned limitation, in practice ECD is often conducted with the assistance of illumination shedding on semiconductor photoelectrodes, namely, the photo‐assisted ECD (PECD). PECD makes use of photogenerated electrons or holes to facilitate the reduction or oxidation the corresponding metal ions, so that the overpotential needed for catalyst nucleation could be decreased; moreover, the deposited catalysts are able to directly contact with the surface sites where photogenerated charges are produced.^99^, ^100^, ^101^, ^102^ Therefore, PECD usually enables better PEC performance to be achieved compared to the ECD conducted in the dark,^103^ due to a better distribution of catalysts and a more intimate interaction with the underlying photoelectrodes. Based on these prominent advantages, PECD has been widely adopted for semiconductor/electrocatalyst coupling in recent years.

**Figure 6:**
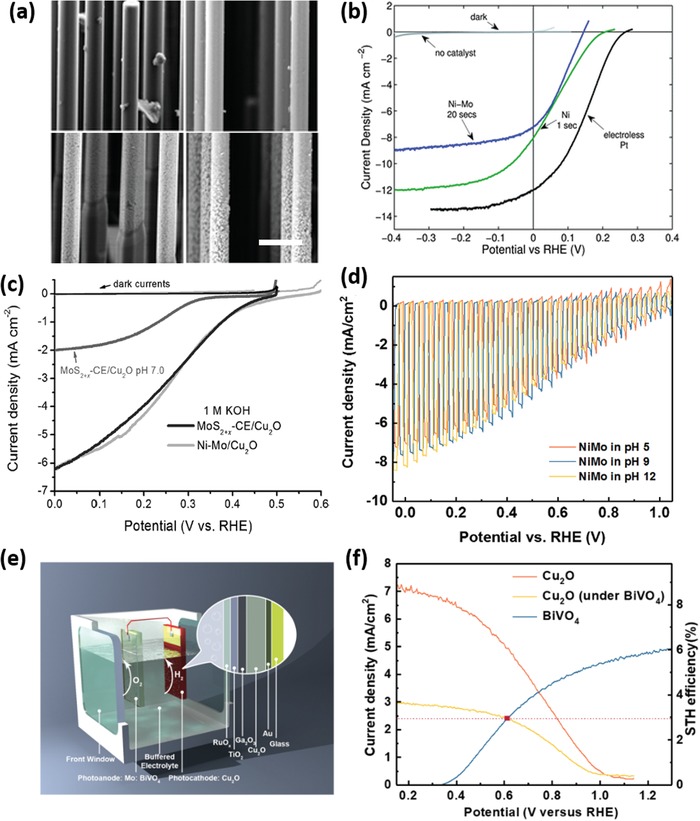
a) SEM images of different catalyst particles on Si microwires. The top (above) and base (below) part of the catalyst films are presented. Left: p^+^‐Si deposited with Ni for 1 s; right: p^+^‐Si deposited with Ni‐Mo for 20 s. The scale bar is 3 µm and applies to all panels. b) Polarization curves of the HER activities of various photocathodes under illumination. Reproduced with permission.^47^ Copyright 2011, The Royal Society of Chemistry. c) LSVs of MoS_2+_ *_x_* and Ni‐Mo‐modified Cu_2_O measured under illumination. Reproduced with permission.^104^ Copyright 2014, Wiley‐VCH. d) Chopped‐light LSVs of Ni‐Mo/Ga_2_O_3_/Cu_2_O photocathodes measured at different pH values. e) Schematic illustration of a tandem PEC cell consisting of a Ni‐Mo/Ga_2_O_3_/Cu_2_O cathode and a BiVO_4_ anode. f) LSV curves of the Ni‐Mo/Ga_2_O_3_/Cu_2_O cathode and modified Mo‐doped BiVO_4_ anode. The cross point indicates the working condition of the two‐electrode system. Reproduced with permission.^105^ Copyright 2018, Springer Nature Publishing AG.

Early studies about PECD of HER or OER catalysts on semiconductor photoelectrodes can date back to 1970s. A variety of metals such as Pt, Pd, Ni, Cu, Rh, and Ru were electrodeposited on different semiconductors to catalytically boost the production of H_2_.^106^ While most early works mainly focused on PECD of catalysts on planar semiconductor photoelectrodes, PECD also demonstrated the capability of depositing catalysts over complex high‐aspect‐ratio semiconductor micro/nanostructures. For instance, Lewis et al. reported galvanostatic and potentiostatic PECD of Ni and NiMo alloy on p‐Si‐µW array photocathodes.^47^ Scanning electron microscope (SEM) imaging and energy‐dispersive spectroscopy analysis confirmed that both Ni and NiMo deposits covered conformally over the entire µW surfaces (**Figure** **6**a). Compared to bare Si‐µWs, both Ni and NiMo‐modified Si‐µW photocathodes showed a substantial positive shift in photocurrent onset (Figure 6b) and a thermodynamically based light‐to‐H_2_ efficiencies of 0.2–0.4%.

In addition to metal/alloy catalysts, MoS_2_ has emerged as a new HER catalyst and been investigated extensively for use in PEC water splitting. Hu's group developed a convenient ECD method to prepare active amorphous MoS*_x_* thin films. The film can be produced from an aqueous solution of (NH_4_)_2_MoS_4_ when the potential was cycled continuously from 0.3 to −0.8 V versus SHE (standard hydrogen electrode, V_SHE_). The amorphous MoS_3_ material was initially formed under oxidative potential, while cycling the potential back to reductive potential promoted the formation of amorphous MoS_2_—the active species, on the surface.^107^ Given the comparatively low electrical conductivity and poor chemical stability of some semiconductor light absorbers, PECD of catalysts usually needs to take place on a conductive and/or protective coating layer deposited on top of photoelectrodes. For instance, Chorkendorff and co‐workers introduced a Ti protection layer which effectively prevented the Si electrode from oxidation during PECD of MoS*_x_*. The thus‐obtained MoS*_x_*‐Ti‐n^+^p‐Si photocathode showed significant anodic shift in the photocurrent onset for HER in comparison to the bare n^+^p‐Si and unprotected MoS*_x_*‐n^+^p‐Si,^108^ and its PEC performance was comparable to that of the Pt‐modified n^+^p‐Si photocathode. Hu's group adopted the PECD method to deposit amorphous MoS*_x_* on Cu_2_O thin films coated in sequence by Al‐doped ZnO (to create a p‐n junction) and TiO_2_ (as protective layer).^104^, ^109^ The PECD enabled the formation of a conformal and continuous MoS*_x_* layer on protected photocathodes, showing a photocurrent density of −5.7 mA cm^−2^ in acid electrolyte (pH = 1.0) and an even higher density of −6.3 mA cm^−2^ in alkaline electrolyte (pH = 13.6), at 0 V_RHE_ under 1 Sun illumination, which favorably compare to Pt‐modified protected Cu_2_O photocathodes but show markedly improved stability (Figure 6c). Moreover, the researchers compared the PEC performance of MoS*_x_*‐modified Cu_2_O to that of Ni‐Mo‐modified photocathodes with the same layered electrode structure,^104^ and found that they exhibited similar performance though Ni‐Mo showed better electrocatalytic HER activity. Grätzel and co‐workers further developed a Ga_2_O_3_‐protected Cu_2_O NW photocathode, where RuO*_x_* and Ni‐Mo were electrodeposited, respectively, under 1 Sun illumination to promote the HER.^105^ Remarkably, a photocurrent onset over +1 V_RHE_ and a photocurrent density of ≈10 mA cm^−2^ at 0 V_RHE_ were achieved with Cu_2_O/Ga_2_O_3_/TiO_2_/RuO*_x_*, rendering it the best oxide‐based photocathode up to now for catalytic generation of H_2_ from sunlight. Even with earth‐abundant Ni‐Mo catalysts, the photoelectrode still showed a similar onset potential (>1 V_RHE_) and a slightly decreased short‐circuit photocurrent density of 8.2 mA cm^−2^ (Figure 6d). Moreover, the photocathode demonstrated outstanding stability in both weak acidic (pH = 5) and weak alkaline (pH = 9) solutions. Furthermore, the authors fabricated an all‐oxide unassisted solar water‐splitting tandem device comprising the as‐fabricated photocathode and a NiFeO*_x_*‐modified Mo‐doped BiVO_4_ photoanode (Figure 6e). Impressively, a solar‐to‐H_2_ conversion efficiency of about 3% was achieved in a carbonate buffer electrolyte (pH = 9, Figure 6f).

Using PECD, other HER catalysts, e.g., MoS*_x_*O*_y_*,^110^ CoMoS*_x_*,^111^ NiCoSe*_x_*,^112^ and CoP,^113^ have also been reported to couple to different photocathodes for light‐driven H_2_ production. In many cases, the catalyst layers are usually directly photodeposited on semiconductor photoelectrodes without introducing an interlayer, and they offer, besides high catalytic activity, good passivation to semiconductors against corrosion given their exceptional electrochemical stability, exhibiting promising bi‐functionality.

**Figure 7:**
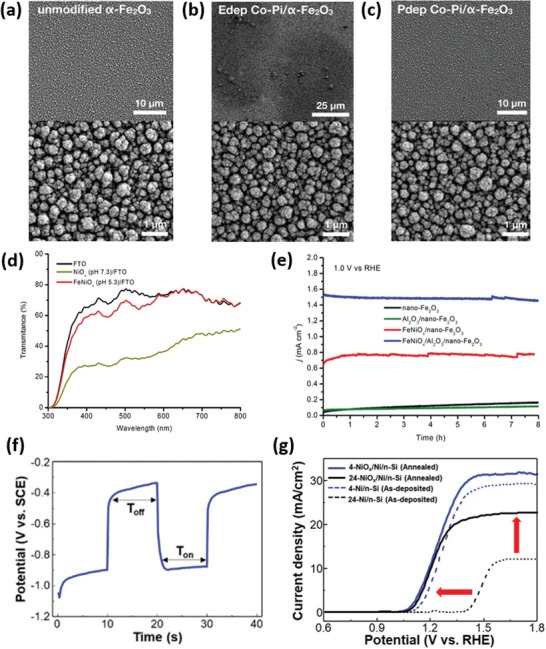
SEM images of a) hematite, b) Co‐Pi/hematite prepared by electrodeposition, and c) Co‐Pi/hematite prepared by photoassisted electrodeposition. Reproduced with permission.^101^ Copyright 2011, The Royal Society of Chemistry. d) Transmittance spectra of NiO*_x_* and FeNiO*_x_* on FTO.^19^ e) Chronoamperometry of FeNiO*_x_*/Al_2_O_3_/nano‐Fe_2_O_3_ and various control samples recorded at 1.0 V_RHE_ under AM 1.5G illumination. Reproduced with permission.^19^ Copyright 2015, The American Chemical Society. f) The potential‐time curve of pulsed electrodeposition of Ni.^121^ g) The PEC performance of different Ni‐modified n‐Si photoanodes. Reproduced with permission.^121^ Copyright 2018, The American Chemical Society.

Aside from photocathodes, PECD was also employed to deposit active OER catalysts to construct efficient photoanodes. A prominent ECD‐derived OER catalyst is the cobalt phosphate (i.e., Co‐Pi).^114^ Co‐Pi can be easily produced from a Co^2+^‐containing phosphate buffer (pH = 7) by applying a positive potential or performing multiple CV scans in a given potential range. The active Co‐Pi is composed of clustered bis‐*µ*‐oxo/hydroxo‐linked Co ions, which is similar to cobaltate compound but possesses molecular dimensions.^115^ This endows Co‐Pi to exhibit self‐healing properties.^114^ Due to the high activity and good stability in a wide pH range, Co‐Pi has been employed as an OER catalyst to couple with a variety of photoanodes, including hematite,^101^, ^116^ BiVO_4_,[qv: 100,103a,117] WO_3_,^118^ Ta_3_N_5_,^119^ Si,^120^ ZnO,^102^ etc., and Co‐Pi coupling usually gives rise to a lower onset potential, a higher photocurrent density, and improved stability. Gamelin and co‐workers systematically studied the performance of Co‐Pi‐modified hematite.^101^ PECD resulted in more uniformly distributed catalyst particles, while distinct aggregation was observed on the photoanode prepared by ECD in the dark (**Figure** **7**a–c). Consequently, the α‐Fe_2_O_3_ modified with PECD Co‐Pi showed more cathodic shift in the onset potential and a higher photocurrent density in comparison to the bare hematite, Co^2+^/hematite, and Co‐Pi/hematite prepared by ECD, highlighting the great advantages of PECD.

In an attempt to extending the “Co‐Pi” family, it was found that tuning the auxiliary electrolyte composition can obtain catalyst films with even higher activity and better stability. Joya et al. developed a Co‐based amorphous film from CO_2_‐saturated bicarbonate solution (Co‐Ci).^122^ In comparison to Co‐Pi, Co‐Ci showed better activity and much improved stability in carbonate and phosphate buffer solutions.^122^ Lee and co‐workers incorporated Co‐Ci onto an H, Mo dual‐doped BiVO_4_ and a WO_3_/BiVO_4_ composite photoanode, respectively.^123^, ^124^ When combining in tandem with a CH_3_NH_3_PbI_3_ perovskite single junction solar cell, the Co‐Ci/H, Mo:BiVO_4_ photoanode exhibited a high PEC water oxidation activity (4.8 mA cm^−2^ at 1.23 V_RHE_) under simulated 1 Sun illumination, which was comparable to that of Co‐Pi modified photoelectrode (5.0 mA cm^−2^ at 1.23 V_RHE_) but showed significantly better stability.^123^ The authors further proved that the Co‐Ci modified photoanode can be used to couple with a Cu cathode for electrocatalytic CO_2_ reduction.^124^ In addition to tuning the supporting electrolyte, ECD allows organic ligands to be introduced, and the formed coordinated metal complex can act as a precursor for the deposition of active catalysts on semiconductor surfaces.^125^ The controllable deposition is conducive to uniform coating of catalysts since metal ions can be slowly released in the ECD process.

The catalyst loading is crucial to the PEC performance of photoelectrodes. As mentioned in Section 2, ideally the deposited catalysts should be optically transparent to avoid competitive parasitic light absorption. In case no additional protective layer is present, the catalyst layer should also be able to passivate semiconductor photoelectrodes against corrosion. ECD, in this respect, allows for easier preparation of the catalysts that meet the above requirements as compared to other wet chemical methods. In 2015, Hu and co‐workers reported the deposition of an optically transparent FeNiO*_x_* catalyst from a near‐neutral acetate electrolyte containing Ni^2+^ and Fe^2+^ by simple anodic linear scan sweeps.^19^ The self‐limiting growth process rendered so low loading mass that the transmittance spectrum of the deposited FeNiO*_x_* film was close to that of FTO glass (Figure 7d). In this case, the parasitic light absorption caused by FeNiO_x_ catalysts can be significantly reduced. In contrast, the NiO*_x_* catalysts deposited under similar conditions showed high absorbance and competitively absorbed the incident light to a great extent. A photoanode comprising nano‐hematite, Al_2_O_3_ overlayer, and ultrathin FeNiO*_x_* catalysts (FeNiO*_x_*/Al_2_O_3_/nano‐Fe_2_O_3_) was found to deliver a stable photocurrent density of 1.5 mA cm^−2^ at 1.0 V_RHE_, while the photoanode without any catalysts showed negligible photocurrent (Figure 7e). When the composite photoanode was coupled with a NiMo HER electrocatalyst and a perovskite solar cell, unassisted water splitting with an STH efficiency of 1.9% was achieved. Recently, Hu's group further extended this strategy, depositing an ultrathin layer of CoFeO*_x_* catalyst on hematite photoanodes.^126^ The CoFeO*_x_*/hematite electrode exhibited a 200 mV cathodic shift in the photocurrent onset potential and a sevenfold increase in stabilized photocurrent density at 1.0 V_RHE_ compared to the catalyst‐free hematite.

In addition to the commonly used CV and galvanostatic/potentiostatic ECD, pulsed ECD was recently utilized as well to deposit catalysts on photoelectrodes. In a typical pulsed ECD, the applied potential or current alternately swifts between two different values. This gives rise to a series of pulses of equal amplitude and duration. Additionally, a hold‐on pulse can be introduced between two sequential pulses, allowing for the recovery of electrolyte concentration at the deposition front. In comparison to conventional CV or galvanostatic/potentiostatic ECD, pulsed ECD may result in more uniformly distributed and denser deposits with lower porosity. Moreover, it helps improve the adhesion of deposit on the substrate and allows for better control over the deposit thickness. Employing pulsed ECD, Rao et al. first reported the integration of Co‐La layered double hydroxide (LDH) catalysts on BiVO_4_ photoanodes.^127^ The co‐deposition of Co and La was performed with alternate cathodic and anodic current pulses, with a hold time of 1 s after each pulse. This offered firm adhesion, uniform composition, and grain size of the Co‐La catalysts. With respect to the bare BiVO_4_, the optimized BiVO_4_/Co‐La LDH showed a significant reduction in the onset potential by 0.53 V and a photocurrent density increase by 33.4 times at 0.6 V_RHE_. Lee et al. fabricated a NiO*_x_*/Ni/n‐Si photoanode with an MIS structure using pulsed ECD followed by a simple thermal annealing (Figure 7f).^121^ The pulsed ECD allows for easy control of the coverage of Ni NPs deposited on Si and therefore affects the PEC performance. The n‐Si photoanode with an optimized coverage of Ni and the formed catalytically active NiO*_x_* shell showed a photocurrent density of 14.7 mA cm^−2^ at 1.23 V_RHE_ and a > 30 mA cm^−2^ saturated photocurrent density due to the enhanced charge separation and transport efficiency (Figure 7g).

**Figure 8:**
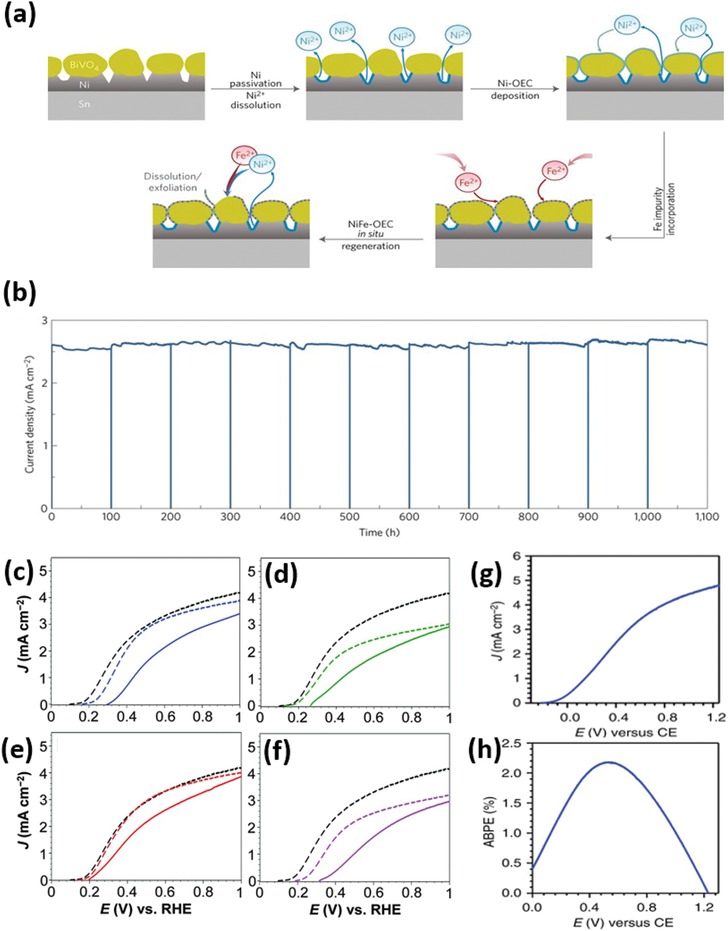
a) Schematic illustration of the mechanism of the in situ generation of NiFeO*_x_* catalyst on BiVO_4_. b) Long‐term stability test of the modified BiVO_4_ photoanode at 0.6 V_RHE_ under AM 1.5G illumination. Reproduced with permission.^128^ Copyright 2016, Springer Nature Publishing AG. *J*–*V* curves of c) BiVO_4_/FeOOH, d) BiVO_4_/NiOOH, e) BiVO_4_/FeOOH/NiOOH, and f) BiVO_4_/NiOOH/FeOOH. The dashed and solid lines represent the photocurrent for sulfite oxidation and water oxidation, respectively. Dashed black lines in (c)–(f) represent the LSV of bare BiVO_4_ in the presence of 1 m Na_2_SO_3_. All the curves were recorded under AM 1.5G illumination in phosphate buffer. Reproduced with permission.^129^ Copyright 2014, The American Association for the Advancement of Science. g) LSV of N‐BiVO_4_/FeOOH/NiOOH. The curve was recorded in a two‐electrode system under AM 1.5G illumination. The counter electrode was Pt. h) The corresponding ABPE (%) derived from (g). Reproduced with permission.^130^ Copyright 2015, Springer Nature Publishing AG.

While for most photocathodes and photoanodes, the electrode architecture is completed once ECD or PECD of electrocatalysts is done, Domen and co‐workers recently reported an interesting work where the OER catalysts could be deposited and regenerated on BiVO_4_:Mo photoanodes through an in situ PECD process.^128^ To this end, they deposited Ni and Sn layers in sequence on particulate BiVO_4_:Mo thin films. During PEC water oxidation, the Ni underlayer was dissolved serving as an ion source to allow for in situ photodeposition of dissolved Ni cations on BiVO_4_:Mo (**Figure** **8**a). The Fe impurities in borate buffer electrolyte were further incorporated into the in situ‐generated NiO*_x_* forming highly active NiFeO*_x_* OER catalysts. Although some catalysts were chemically dissolved or physically lost connection with BiVO_4_:Mo during long‐term operation, they could be regenerated quite fast. Therefore, ultrahigh stability of 1100 h continuous operation could be achieved at a low bias (Figure 8b).^128^ This study suggests that adding a small amount of metal impurities in electrolyte might be able to suppress the dissolution of catalysts since the dissolution and the regeneration of catalysts are in equilibrium. In this way, the stability of photoelectrodes could be markedly improved.

As mentioned above, loading a highly active electrocatalyst on a semiconductor photoelectrode does not surely lead to high PEC performance. The interaction between the semiconductor and the catalysts plays an important role, and poor interaction typically results in deteriorated PEC performance even if the loaded catalyst itself has an outstanding dark electrocatalytic activity. For instance, Kim and Choi photoelectrodeposited FeOOH and NiOOH OER catalysts on BiVO_4_ photoanodes and studied the influence of these two catalysts on the PEC performance.^129^ They found that although NiOOH is more catalytically active than FeOOH, BiVO_4_/NiOOH showed much lower photocurrent density than BiVO_4_/FeOOH. Using Na_2_SO_3_ as a hole scavenger, the authors measured the yield of surface‐reaching holes of bare BiVO_4_ (black dashed lines in Figure 8c–f), and compared it to the PEC water oxidation *J*–*V* curves of BiVO_4_/FeOOH and BiVO_4_/NiOOH (solid lines in Figure 8c,d). They concluded that FeOOH can better reduce interface recombination, because it forms much better interface with BiVO_4_ as compared to NiOOH. Furthermore, they found that NiOOH may render a favorable Helmholtz layer potential drop leading to an early photocurrent onset, besides its high catalytic activity. Based on these findings, the researchers designed a dual‐layer catalyst by sequential PECD of FeOOH and NiOOH on BiVO_4_ (BiVO_4_/FeOOH/NiOOH). Impressively, this unique catalyst structure enabled by a two‐step PECD is able to harvest a significant portion of surface‐reaching holes for PEC water oxidation (Figure 8e). Accordingly, the BiVO_4_/FeOOH/NiOOH photoanode exhibited a photocurrent density of 2.73 mA cm^−2^ in phosphate buffer (pH = 7) at a low potential of 0.6 V_RHE_. In contrast, the BiVO_4_/NiOOH/FeOOH resulting from a reverse two‐step PECD showed the worst PEC water oxidation performance due to the undesirable interfacial contact and poor surface charge transfer kinetics (Figure 8f). The same group further extended the multi‐step deposition process to a N‐doped BiVO_4_,^130^ where apart from the dual PECD of FeOOH and NiOOH, a third ECD process of NiOOH was carried out on top of N‐BiVO_4_/FeOOH/NiOOH, in order to cover any bare BiVO_4_ or FTO surfaces exposed to the electrolyte. This three‐step catalyst deposition enabled more uniform distribution of the catalysts, and consequently an onset potential as low as 0.25 V_RHE_ and an applied bias photon‐to‐current efficiency (ABPE) exceeding 2% were achieved (Figure 8g,h).

#### 3.2.2 ELD

ELD is an auto‐catalytic plating process where the substrate develops a potential in the electrolyte containing metal ions, reducing agent, and other components. Unlike ECD, ELD occurs without the application of an external bias. ELD of metals can occur spontaneously if the redox potential of the metal couple (e.g., Pt^2+^/Pt) is higher than that of both the semiconductor surface and the hydrogen evolution. In order to increase the charge carrier density for the redox reaction, illumination is needed in some cases.

**Figure 9:**
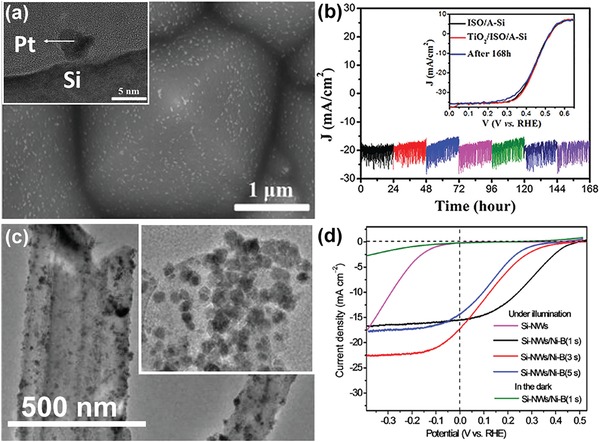
a) SEM image showing uniform distribution of fine Pt NPs on the surface of n^+^np^+^‐Si photocathode prepared by ELD in isopropanol diluted (with 5% H_2_O) solution containing 2 m HF and 1 × 10^−3^ m K_2_PtCl_6_·6H_2_O. Inset: TEM image showing the interface between Pt NPs and Si. b) PEC stability measurements for the TiO_2_‐protected Si photocathodes coated with ELD fine Pt NPs, performed at 0.4 V_RHE_. Inset: the *J*–*V* curves of different photocathodes measured after the 168 h PEC test. Reproduced with permission.^132^ Copyright 2018, The Royal Society of Chemistry. c) TEM image showing the morphology of ELD Ni‐B NPs distributed on SiNWs. d) Polarization curves of SiNWs/Ni‐B photocathodes prepared with different ELD durations, measured in phosphorous buffer solution (pH = 7). Reproduced with permission.^133^ Copyright 2016, The American Chemical Society.

ELD has been used to deposit catalysts on semiconductor photoelectrodes for a long time.^131^ Recently, Shen's group investigated how the electrolyte composition affects the ELD of Pt on Si and the PEC performance of the resulting photocathodes.^132^ They found that ELD of Pt with an aqueous solution containing 2 m HF and 1 × 10^−3^ m K_2_PtCl_6_·6H_2_O on n^+^np^+^‐Si, as researchers usually do, led to the formation of large and nonuniform Pt NPs on the Si surface. In contrast, if isopropanol‐diluted solution of 2 m HF and 1 × 10^−3^ m K_2_PtCl_6_·6H_2_O is used, uniformly distributed fine Pt NPs (5–10 nm) appeared with a high density on the Si surface (**Figure** **9**a). Furthermore, the authors demonstrated that Pt NP size and distribution could be tuned by adjusting the water/isopropanol ratio. The optimized platinized n^+^np^+^‐Si photocathode coated with a TiO_2_ protection layer showed a high efficiency of 11.5% and outstanding stability at 0.4 V_RHE_ over 7 days (Figure 9b). Oh et al. lately reported ELD of Pt on p‐GaAs photocathodes, and compared the PEC performance of GaAs/ELD‐Pt to that of GaAs modified by e‐beam‐evaporated Pt catalyst layers.^48^ They found that although ELD Pt was structurally inhomogenous, the distribution minimized the recombination of excited carriers because of the pinch‐off effect. Moreover, ELD Pt was also optically more transparent than the e‐beam‐evaporated Pt catalyst layer. Accordingly, GaAs/ELD Pt achieved better PEC performance.

For many metal ELD processes, HF is added to assure continuous metal growth. However, the addition of HF makes the control over the density and morphology of deposited NPs difficult. To overcome the limitation, ELD of metals on semiconductor photoelectrodes in fluoride‐free solution has been developed. In this process, the growth of metal NPs is self‐limiting, and it is ceased when the electron transfer between the semiconductor and the metal cations is blocked by the formed metal oxide layer that cannot be dissolved. For example, Meriadec et al. utilized ELD to deposit Pt and Au on hydrogen‐terminated p‐Si surfaces in aqueous solution only containing the corresponding metal salt, which resulted in uniform small NPs evenly distributed on p‐Si.^134^ Furthermore, they demonstrated the possibility of creating Au and Pt bi‐metallic micropatterns on the p‐Si surface. Their preliminary results showed that thus‐obtained photocathodes exhibited a significant decrease in photocurrent onset potential as compared to the bare Si. Furthermore, considering the unique plasmon‐enhanced light absorption of Au and high catalytic activity of Pt, ELD of these two metals on the same semiconductor photoelectrode offers the possibility of simultaneously tuning the light absorption and surface charge transfer. Zheng's group recently also reported ELD of Ni thin films on n‐Si in a fluoride‐free solution without any reducing agent.^135^ In this case, Si serves as a reductant for the growth of Ni. Interestingly, the authors found an intermediate porous SiO_2_ layer formed between the Si substrate and the top Ni layer, where metallic Ni was embedded to act as conductive pathways allowing the photogenerated charge carriers to be effectively transferred to the electrolyte. The optimized Ni/n‐Si photoanodes showed an onset potential of 1.09 V_RHE_ and a saturated photocurrent density of 27.5 mA cm^−2^ under AM 1.5G illumination at pH = 14. Furthermore, they demonstrated that this Ni ELD process enabled the formation of a uniform Ni layer over a 4 in. Si wafer, showing great potential for large‐scale fabrication of Si photoanodes.

In addition to metal/alloy catalysts, compound electrocatalysts can also be deposited on semiconductors using ELD. For example, Sun's group reported the electroless plating of Ni‐B and Co‐B HER catalysts on p‐Si NW array photocathodes.^133^ The plating solution contained Ni^2+^ or Co^2+^ coordinated by organic ligands (ethanediamine for Ni‐B and glycine for Co‐B), which was prepared in an ice bath. The cooled precursor solution was then reduced by NaBH_4_ under alkaline conditions. After the solution was heated to a desired temperature, the p‐Si NW electrode was dipped into the solution for only 1–15 s to complete deposition. The deposition resulted in Ni‐B and Co‐B NPs uniformly distributed along the entire NWs from tip to base (Figure 9c). The optimized electrode exhibited a short‐circuit photocurrent density up to 19.5 mA cm^−2^ in neutral buffer solution under simulated AM 1.5G illumination (Figure 9d). Moreover, the onset potential of cathodic photocurrent was positively shifted to 0.30–0.45 V_RHE_. Remarkably, the half‐cell STH efficiencies reached 2.53% for p‐Si‐NWs/Co‐B and 2.45% for p‐Si‐NWs/Ni‐B, comparable to that p‐Si NW photocathode modified by Pt catalysts. Ni‐P is another emerging electrocatalyst that can be used to catalyze both HER and OER.^136^ Although ELD of Ni‐P on Si substrates was already reported,^137^ PEC performance of semiconductors coupled with ELD Ni‐P catalysts has not been investigated yet.

#### 3.2.3 Dip Coating

Dip coating involves precisely controlled immersion and withdrawal of a substrate into and out of a precursor solution for the purpose of depositing a layer of material. This method has been widely adopted in chemical industry and academic research to create various kinds of thin films. To deposit a catalyst on a semiconductor photoelectrode, typically the photoelectrode needs to be immersed into the solution containing the coating material at a constant speed to avoid jitter. This is followed by dwelling in the solution for a certain period of time. After that, the electrode needs to be taken out carefully, and the solvent is allowed to evaporate forming a thin layer on the photoelectrode. In some cases, the unbounded or weak‐bounded precursors should be washed away. The above steps can be repeated several times to obtain a catalyst film with desired thickness. Dip coating is a suitable method for semiconductor/electrocatalyst coupling when the precursors or catalysts have strong adhesion to photoelectrodes. Due to the low energy consumption and the easy operation, dip coating is extensively adopted to deposit various catalysts on photoelectrodes for PEC water splitting. Moreover, it holds great potential for large‐scale fabrication.

**Figure 10:**
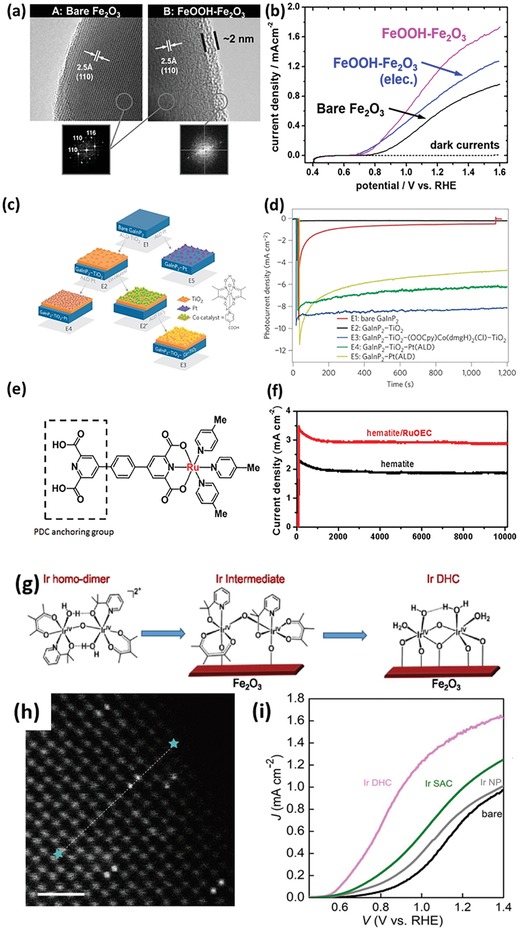
a) The HRTEM images and the corresponding selected area electron diffraction patterns of hematite and the deposited amorphous FeOOH.^138^ b) Comparison of *J*–*V* curves of FeOOH/α‐Fe_2_O_3_ (dip coating), FeOOH/α‐Fe_2_O_3_ (photoassisted electrodeposition), and bare α‐Fe_2_O_3_. Reproduced with permission.^138^ Copyright 2016, Wiley‐VCH. c) Fabrication process of the GaInP_2_/TiO_2_/CoHEC/TiO_2_ photocathode.^139^ d) Chronoamperometry of the GaInP_2_/TiO_2_/CoHEC/TiO_2_ photocathode and control samples recorded at 0 V_RHE_ under AM 1.5G illumination. Reproduced with permission.^139^ Copyright 2015, Springer Nature Publishing AG. e) Structure of molecular Ru catalysts modified by PDC group.^140^ f) *J*–*V* curves of Ru catalyst‐modified α‐Fe_2_O_3_ and the bare α‐Fe_2_O_3_. Reproduced with permission.^140^ Copyright 2015, Wiley‐VCH. g) The process of generation of Ir double atom catalysts on hematite.^141^ h) Aberration‐corrected HAADF‐STEM image of Ir double atom catalysts‐modified hematite.^141^ Scale bar: 1 nm. i) Comparison of *J*–*V* curves of Ir double atom, Ir single atom, Ir NP‐modified and bare hematite electrodes under AM 1.5G illumination. Reproduced with permission.^141^ Copyright 2018, The National Academy of Sciences, USA.

As early as in 2006, Grätzel's group reported the fabrication of Si‐doped hematite photoanodes modified with a monolayer of Co^II^ by dip coating the electrode in 10 × 10^−3^ m Co(NO_3_)_2_.^142^ This simple cobalt treatment resulted in an 80 mV cathodic shift of photocurrent onset and a photocurrent density increase from 2.2 to 2.7 mA cm^−2^ at 1.23 V_RHE_. Control experiments suggested that the active catalyst was the adsorbed Co^II^ monolayer rather than Co(OH)_2_ and cobalt oxide. Furthermore, the post‐treatment that led to aggregation of atomic cobalt sites would decrease photocurrent. It is worth noting that the monolayer catalyst cannot be readily created by other wet chemical methods, and this emphasizes the uniqueness of dip coating in precise control of catalyst layer thickness. Zou and co‐workers deposited Co_3_O_4_ and Co(OH)*_x_* NPs on Ta_3_N_5_ photoanodes by an impregnation method.^143^ They prepared colloidal Co(OH)*_x_* solution by adding NaOH into an aqueous solution containing Co^2+^ ions. The Ta_3_N_5_ photoanode was then immersed into the as‐prepared Co(OH)*_x_* colloidal solution followed by washing and drying. The loaded Co(OH)*_x_* was further transformed to Co_3_O_4_ by a simple annealing process. Co(OH)*_x_*/Ta_3_N_5_ showed a slightly higher photocurrent density at high potentials, but Co_3_O_4_/Ta_3_N_5_ exhibited remarkably improved stability. Recently, Lee et al. demonstrated a simple procedure for depositing an ultrathin FeOOH catalyst layer on hematite photoanodes.^138^ They simply immersed the hematite thin film in a hot aqueous solution containing Fe^3+^ to let an ultrathin amorphous FeOOH (≈2 nm) grow on the electrode surface (**Figure** **10**a). The uniform and highly conformal coating of the ultrathin FeOOH not only improved the water oxidation kinetics, but also passivated the surface states of hematite, leading to an increase of water oxidation photocurrent density by a factor of 2 at 1.23 V_RHE_ and a 0.12 V cathodic shift in onset potential. By contrast, the control sample decorated with FeOOH by photoassisted electrodeposition showed an increase in photocurrent density only by 36% (Figure 10b). This result again underpins the prominent advantage of the simple dip‐coating method for semiconductor/electrocatalyst coupling.^138^

Dip‐coating method was also widely used to couple molecular electrocatalysts to photoanodes or photocathodes. In this case, introducing oxygen or nitrogen‐containing functional groups is necessary to immobilize the molecular catalysts on semiconductors. To do so, the photoelectrode is usually dipped in a solution containing functionalized molecular catalysts for a certain period of time, during which the functional groups are reacting with the surface –OH group of the photoelectrodes, forming either coordination or covalent bonds.^139^, ^140^, ^144^, ^145^ Molecular catalysts containing –COOH or –PO_3_H_2_ groups were already reported to anchor to different semiconductor photoelectrodes.^139^, ^145^ However, they were not stable in neutral and basic aqueous solutions because of the competitive substitution reaction by OH^−^ or anions from the buffer. To overcome this limitation, Turner's group introduced an amorphous TiO_2_ protective layer and obtained a stable molecular‐catalyst‐modified p‐GaInP_2_ photocathode.^139^ The surface‐protected GaInP_2_ was first dipped in a solution containing –COOH‐modified Co‐based HER molecular catalyst, and after adsorption the surface was protected by another thin layer of amorphous TiO_2_ coating (Figure 10c). The resulting photocathode delivered a photocurrent density of 9 mA cm^−2^ at 0 V_RHE_ under 1 Sun illumination at pH = 13, showing better performance than that of the Pt decorated GaInP_2_ electrode (Figure 10d). Moreover, the electrode was capable of steadily producing H_2_ during a 20 h period, with a turnover number of 1 39 000.

Sun's group developed a molecular Ru catalyst with 2,6‐pyridinedicarboxylic acid (PDC) as a novel anchoring group that showed impressive stability when deposited on hematite (Figure 10e).^140^ No significant desorption was observed even immersing the modified electrode in 1 m KOH. Under 1.4 V_RHE_ bias, the photocurrent density remained at around 3 mA cm^−2^ for more than 10 000 s (Figure 10f).

It is worth mentioning that removing the fragile organic ligands of anchored molecular catalysts may result in atomically dispersed catalysts including single‐atoms and/or clusters with precise number of atoms. These atomically dispersed catalysts usually exhibit higher stability than the anchored molecular catalysts due to the lack of unstable organic ligands and the stronger interaction with the support (i.e., photoelectrodes). Wang et al. reported a hematite photoanode modified with Ir double‐atom catalysts obtained from anchored di‐nuclear molecular Ir precursors.^141^ The organic ligands were removed by a simple UV‐assisted photochemical route (Figure 10g). The double‐atom species gave rise to better PEC performance in comparison to Ir single‐atom and Ir NPs in near neutral solution (pH = 6.0, 0.1 m KNO_3_) (Figure 10h,i). In addition, compared to the previously reported hematite electrode decorated with di‐nuclear molecular Ir catalysts, the double‐atom Ir catalyst modified hematite exhibited significantly higher operational stability.^141^, ^144^

#### 3.2.4 SILAR

SILAR is a simple and cost‐effective method to grow polycrystalline or epitaxial thin films of water‐insoluble ionic or iono‐covalent C_m_A_n_ type compounds by interfacial chemical reaction between adsorbed C^n+^ cations and A^m−^ anions. The process involves an alternate immersion of the substrate in a solution containing a soluble salt of the cation (C^n+^) and then in a solution containing a soluble salt of the anion (A^m−^). After each reaction cycle, the substrate needs to be rinsed in high‐purity deionized water to remove unreacted ions. The thickness of the deposited films can be controlled by the number of reaction cycles.^146^

**Figure 11:**
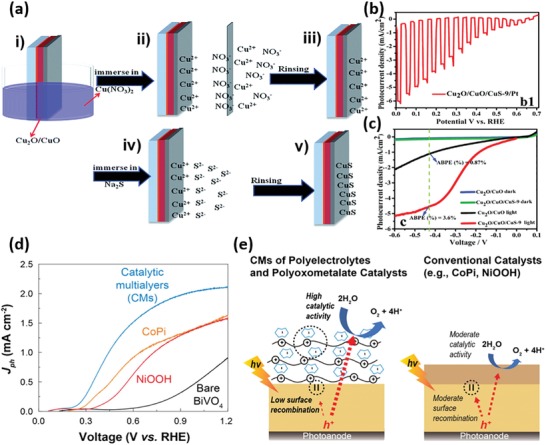
a) Fabrication of CuS on Cu_2_O/CuO photocathodes via a SILAR process.^148^ b) Chopped‐light *J*–*V* curve of Cu_2_O/CuO/CuS‐9‐Pt.^148^ c) *J*–*V* curves of Cu_2_O/CuO/CuS‐9‐Pt and the control samples. Reproduced with permission.^148^ Copyright 2016, The Royal Society of Chemistry. d) *J*–*V* curves of the multilayer Co‐POM‐modified BiVO_4_ in comparison with the Co‐Pi and NiOOH‐modified BiVO_4_ and the bare BiVO_4_.^150^ e) Scheme of Co‐POM and conventional catalysts‐modified BiVO_4_ and the illustration of the mechanisms of charge transport. Reproduced with permission.^150^ Copyright 2019, The American Chemical Society.

SILAR has been extensively employed previously for sensitizing semiconductor light absorbers to improve the photovoltaic performance (e.g., enhanced light absorption).^147^ It was recently also used to deposit catalysts on semiconductor photoelectrodes to boost the PEC performance. For example, Hwang's group reported a Cu_2_O/CuO/CuS heterostructured photoelectrode, where CuS, as an electrocatalyst, was loaded on the Cu_2_O/CuO heterostructure via a SILAR approach.^148^ The authors used Cu(NO_3_)_2_ and Na_2_S as the source of Cu^2+^ cations and S^2−^ anions, respectively, and found that only nine successive SILAR cycles could yield an optimal performance for light‐driven H_2_ evolution (**Figure** **11**a). Moreover, they showed that with co‐deposited Pt catalysts the Cu_2_O/CuO/CuS photoelectrode could accomplish a photocurrent density of 5.7 mA cm^−2^ at 0 V_RHE_ and an ABPE of 3.6% (Figure 11b,c). The catalytic role of SILAR‐deposited CuS was also confirmed recently in TiO_2_/CuS photoelectrodes for PEC water splitting.^149^

Transition metal hydroxides and oxyhydroxides are promising OER catalysts and had been attempted to deposit on hematite photoanodes using SILAR. For instance, by dropping Ni(NO_3_)_2_ and NaOH on Ti‐doped hematite alternately, Li's group prepared a Ti‐Fe_2_O_3_/Ni(OH)_2_ electrode.^151^ Although Ni(OH)_2_ is generally accepted to be an active OER catalyst, the authors concluded that Ni(OH)_2_ thus deposited primarily serves as a hole storage layer to improve the charge transfer across the interface between hematite and the IrO_2_ catalysts deposited on top of Ni(OH)_2_. Baxter and co‐workers managed to deposit a FeOOH overlayer on Ti‐doped hematite thin films using SILAR, which significantly increased the hole transfer efficiency to ≈100%. However, after careful analyses, they pointed out that FeOOH played no catalytic role in PEC water oxidation, although electrocatalytically it is a good OER catalyst. Mullins's group observed a decreased photocurrent when depositing Ni(OH)_2_ on hematite. To solve this problem, they used SILAR to deposit a Ce‐doped Ni(OH)_2_ layer on hematite, based on the consideration that Ce may catalytically tune the oxidation state of the Ni species to the most OER‐active state and reduce the binding energy of OER intermediates at Ni centers.^152^ Indeed, the hematite/NiCeO*_x_* showed improved PEC performance than both the bare hematite and hematite/Ni(OH)_2_.

Interestingly, not only heterogenous catalysts but POM (polyoxometalates) based molecular catalysts can also be stabilized on photoelectrodes by SILAR. Recently, Bae et al. reported the fabrication of a BiVO_4_ photoanode decorated with catalytic multilayers (CMs) composed of a Co‐based POM catalyst and poly(diallydimethylammonium chloride) (PDDA) polyelectrolyte.^150^ The electrode was prepared by alternately dipping in the PDDA and POM solutions followed by washing. The process was repeated for the desired cycles. The optimized CM‐modified BiVO_4_ exhibited better PEC performance than BiVO_4_ deposited by conventional Co‐Pi or NiOOH catalysts (Figure 11d). The authors attributed the better performance to the fact that CMs can improve the kinetics of both the photogenerated charge carrier separation/transport in bulk BiVO_4_ and water oxidation at the electrode/electrolyte interface, while the conventional OER catalysts were mostly effective in the former and less effective in the latter (Figure 11e). In this case, the separated charge carriers are efficiently transported to POM catalysts via a hopping mechanism, which is reminiscent of natural photosynthetic systems.

#### 3.2.5 Hydrothermal/Solvothermal Treatment

Hydrothermal/solvothermal reaction is one of the most commonly used wet chemical methods for materials synthesis, which is usually carried out in a sealed autoclave reactor in the presence of reactants and/or mineralizers in an aqueous (hydrothermal) or nonaqueous (solvothermal) solution under high temperature and high pressure conditions. In comparison to other wet chemical methods, hydrothermal/solvothermal reaction allows for the deposition of catalysts with a higher degree of crystallinity and less surface defects, which may lead to better charge transfer kinetics. Given the reactive nature, metal/alloy catalysts are difficult to be grown through hydrothermal/solvothermal reactions, and the semiconductor photoelectrode should be thermally stable to avoid dissolution and/or reacting with the solution which may change its intrinsic semiconducting character. For these reasons, hydrothermal/solvothermal reaction has by far only been applied to few immobilized semiconductor/electrocatalyst PEC systems, which will be summarized in the following.

As mentioned above, MoS_2_ is an emerging HER catalyst and has been deposited on semiconductor photoelectrodes using different methods. Hydrothermal/solvothermal growth of MoS_2_ was mainly conducted on metal sulfide photocathodes such as CdS,^153^ ZnIn_2_S_4_,^154^ or metal oxide photoanodes including TiO_2_,^155^ ZnO,^156^ and BiVO_4_.^157^ For example, using Na_2_MoO_4_·2H_2_O and urea as precursors, Liang and co‐workers hydrothermally grew MoS_2_ NSs on Cu‐doped CdS NRs.^153^ They found that 5% loading of MoS_2_ could substantially improve the HER rate, and they attributed the improvement to rapid separation and transfer of photogenerated charge carriers enabled by MoS_2_ loading and Cu^2+^ doping. Chai et al. used a solvothermal method to load MoS_2_ on ZnIn_2_S_4_ microspheres,^154^ and verified efficient charge transfer and separation in the MoS_2_/ZnIn_2_S_4_ composite electrodes. Although MoS_2_ was intensively studied as an HER catalyst, it was recently reported to be active for OER as well.^66^, ^158^ Recent attempts to loading MoS_2_ on ZnO NR array and BiVO_4_ IO photoanodes via hydrothermal treatment demonstrated that MoS_2_ played multiple roles in the enhancement of PEC performance: it not only cathodically shifts the photocurrent onset and increases the photocurrent density, but also enhances the absorption of visible light.^156^, ^157^ However, the oxidation of MoS_2_ under water oxidation conditions should be taken in account for long‐term operation. Besides MoS_2_, other sulfides such as Ni_3_S_2_ were also hydrothermally/solvothermally deposited on semiconductors to improve the PEC performance.^159^

Another category of electrocatalysts that are coupled to semiconductor photoelectrodes via a hydrothermal or solvothermal approach are transition metal oxides and LDHs. Nam et al. used a stepwise hydrothermal treatment to fabricate a hierarchical hematite photoanode composed of pure Fe_2_O_3_ underlayer, Ti‐doped Fe_2_O_3_ NWs, and β‐FeOOH catalyst.^160^ The underlayer significantly increased the photocurrent density and decreased the onset potential. β‐FeOOH catalyst further improved the photocurrent but the onset potential was positively shifted due probably to the increased flat‐band potential. Wong and co‐workers loaded Fe_2_TiO_5_ on hematite NR photoanodes using an isopropanol solution containing Fe(acac)_3_ and titanium tetra‐isopropoxide as precursors.^161^ Although Fe_2_TiO_5_ itself is not a good electrocatalyst for OER, it can improve the charge separation and the efficiency of hole injection of the hematite when the Fe_2_O_3_/Fe_2_TiO_5_ heterojunction is formed. In this way, the Fe_2_O_3_/Fe_2_TiO_5_ photoanode showed a photocurrent density of ≈1.4 mA cm^−2^ and high surface charge separation efficiency of 85% at 1.23 V_RHE_. Using a similar solvothermal approach, Gao et al. further deposited Fe_2_TiO_5_ on BiVO_4_.^162^ Compared to bare BiVO_4_, BiVO_4_/Fe_2_TiO_5_ showed a 300 mV cathodic shift in onset potential and three times enhancement in photocurrent at 1.23 V_RHE_. Comprehensive electrochemical and optical characterization confirmed that the enhanced PEC performance originated mainly from the surface passivation effect of Fe_2_TiO_5_.

**Figure 12:**
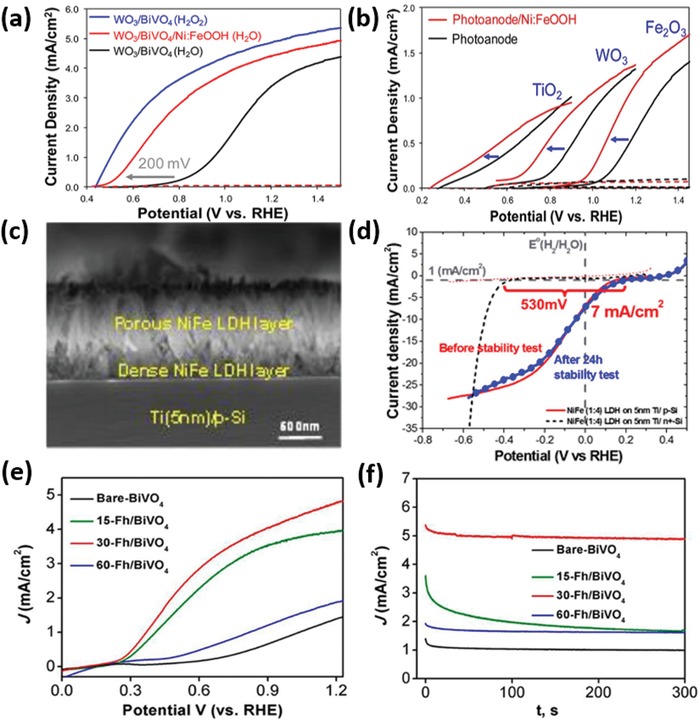
a) The *J*–*V* curve of Ni:FeOOH‐modified WO_3_/BiVO_4_ in comparison with that of bare WO_3_/BiVO_4_ electrode.^164^ b) Comparison of *J*–*V* curves of various Ni:FeOOH‐modified photoanodes. Reproduced with permission.^164^ Copyright 2016, The American Chemical Society. c) Cross‐sectional SEM image showing the growth of NiFe LDH on p‐Si.^165^ d) *J*–*V* curves of NiFe LDH/p‐Si (red trace) compared to the bare p‐Si (black dashed trace). The *J*–*V* curve after stability test is given for comparison (blue trace). Reproduced with permission.^165^ Copyright 2017, The American Chemical Society. e) *J*–*V* curves of BiVO_4_ with different Fh loadings. f) *J*–*t* curves of BiVO_4_ with different Fh loadings recorded at 1.23 V_RHE_. Reproduced with permission.^166^ Copyright 2017, The American Chemical Society.

NiFe LDH has been reported to show higher OER activity than FeOOH and NiOOH, and can be readily obtained by hydrothermal or solvothermal treatment. Schmuki and co‐workers hydrothermally grew NiFe LDH on a Ta_3_N_5_ NR array photoanode,^163^ and observed a photocurrent density increase to 1.7 mA cm^−2^ at 1.23 V_RHE_ in 1 m KOH. Co‐depositing with Co(OH)*_x_* and Co‐Pi, the photocurrent density could be further improved to 6.3 mA cm^−2^. Moreover, the NiFe LDH dramatically reduced the photo‐corrosion effects and could help maintain a stable photocurrent (90% of initial value after 2 h). Zheng's group employed a hydrothermal method to deposit Ni‐doped FeOOH (Ni:FeOOH) on the surface of WO_3_/BiVO_4_ NWs, which generated a photocurrent density of 4.5 mA cm^−2^ and a charge transfer efficiency of 91% at 1.23 V_RHE_ under AM 1.5G illumination (**Figure** **12**a).^164^ Additionally, they demonstrated that hydrothermal growth of Ni:FeOOH is a general and cost‐effective method and can be applied to various photoanodes including WO_3_, α‐Fe_2_O_3_, TiO_2_ NWs, BiVO_4_ films, and Si wafers (Figure 12b).^164^ In all cases, the Ni:FeOOH catalyst negatively shifted the onset potentials, due to its open tunnel structure, tuneable doping concentration, and high‐quality contact with the underlying photoabsorber. The same group further hydrothermally coated NiFe LDH on p‐Si photocathodes.^165^ A thin layer of Ti (≈5 nm) was predeposited on p‐Si to protect it from oxidation under hydrothermal conditions and to improve the adhesion between LDH and the photocathode. The NiFe LDH acted as both an HER catalyst and a protective layer (Figure 12c). In addition, the NiFe LDH/Ti formed a type‐II heterojunction with the underneath p‐Si substrate, which led to effective electron injection and hole blocking for HER. As a result, in 1 m KOH the NiFe LDH‐protected/catalyzed p‐Si photocathode showed a photocurrent density of 7 mA cm^−2^ at 0 V_RHE_, an onset potential of ≈0.3 V_RHE_ (Figure 12d), and good stability at 10 mA cm^−2^ for at least 24 h under AM 1.5G illumination.

Besides metal sulfides, oxides, and LDHs, some emerging or complex catalysts were also reported to be able to couple to semiconductor photoelectrodes via hydrothermal/solvothermal treatment. For example, Xia et al. recently reported hydrothermal deposition of CoP NSs on porous BiVO_4_,^167^ which resulted in a cathodic shift in onset potential by more than 220 mV and an improved photocurrent density of 4.0 mA cm^−2^ at 1.23 V_RHE_, superior to that of porous BiVO_4_ modified by Co_3_O_4_ and Co‐Pi catalysts. Furthermore, the authors compared the PEC performance of the BiVO_4_ modified by hydrothermally loaded CoP NSs to that of the BiVO_4_ modified by drop‐cast CoP NSs, and found that the former markedly outperformed the latter. Co_3_(OH)_2_(HPO_4_)_2_ was also grown on LaTiO_2_N photoanodes using a hydrothermal process,^168^ which enhanced the PEC OER activity by 3.5 times when compared to the bare LaTiO_2_N electrode.

As discussed above, optimization of the catalyst loading or thickness is important to achieve high PEC performance. Hydrothermal/solvothermal treatment, in this regard, allows for facile tuning of the catalyst loadings by controlling the reaction time. For example, Li et al. used hydrothermal method to deposit Fh on a worm‐like nanoporous BiVO_4_.^166^ The authors found that a hydrothermal reaction time of 30 min (30Fh‐BiVO_4_) yielded a stable photocurrent of 2.7 mA cm^−2^ at 0.61 V_RHE_ and an ABPE of 1.81% (Figure 12e), showing the best PEC performance. The dispersed Fh NPs (15Fh‐BiVO_4_) produced with a shorter reaction time could accelerate hole transfer for water oxidation, but the resulting photoanode suffered from poor stability (Figure 12f). In contrast, a thicker layer of Fh (60Fh‐BiVO_4_) improved the stability by suppressing photo‐corrosion and surface CR, but the photocurrent density of 60Fh‐BiVO_4_ was small due to the reduced hole transfer rate.

#### 3.2.6 Droplet‐Based Deposition

Droplet‐based deposition, including drop‐casting, spin‐coating, and spray coating, are straightforward and commonly used methods to couple electrocatalysts to semiconductor photoelectrodes. While they are essentially very similar, the major difference lies in the means of dispersing solutions on a substrate of interest. Drop casting involves the impingement of a solution drop onto a substrate, which usually leads to a nonuniform thin film after solvent evaporation. In contrast, spin coating takes advantage of centrifugal force to spread the coating solution onto a rotating substrate, which allows for the formation of comparatively uniform thin films and better control over the film thickness by tuning the rotating speed, viscosity, and concentration of the coating solution. Spray coating refers to a process in which the (heated) solution is sprayed onto a substrate. In case NP suspension is used, ultrasonic vibration in most cases needs to be applied to avoid the clogging of nozzles. Making use of the droplet‐based deposition methods, the semiconductor/electrocatalyst coupling can be realized either by direct drop/spin/spray coating of a suspension containing active electrocatalysts onto the semiconductor photoelectrode, or by loading catalyst precursors onto the photoelectrode, followed by converting the precursors into active catalysts through post‐treatment. In the former case, a post‐treatment or a polymeric binder is often necessary as well to improve the adhesion of catalysts to the semiconductor surface and thereby the operational lifespan of photoelectrodes.

**Figure 13:**
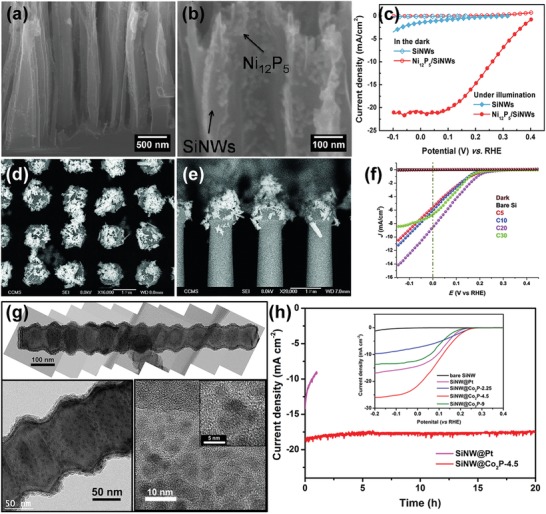
a,b) SEM images showing the morphology of Ni_12_P_5_/SiNWs photocathodes. c) Photocurrent and dark current of Ni_12_P_5_/SiNWs photocathode and bare SiNWs. Reproduced with permission.^170^ Copyright 2014, The American Chemical Society. d) Top and e) cross‐sectional views of CoSe_2_ NR‐decorated Si‐µW array photocathodes. f) Linear sweep voltammograms of Si/CoSe_2_ photoelectrodes with different loadings of CoSe_2_. Reproduced with permission.^171^ Copyright 2015, Wiley‐VCH. g) TEM images showing conformal and continuous deposition of the Co_2_P catalyst layer on SiNWs. h) PEC stability tests of SiNW@Co_2_P and SiNW@Pt photocathodes conducted at 0 V_RHE_ under AM 1.5G illumination in 0.5 m H_2_SO_4_. Inset: LSV curves of SiNW@Co_2_P photocathodes with different Co_2_P loadings. Reproduced with permission.^182^ Copyright 2018, Springer Nature Publishing AG.

Different nanostructured catalysts have been attempted to directly cast on a variety of semiconductor photoelectrodes. In 2011, Chorkendorff and co‐workers reported that drop‐casting molecular Mo_3_S_4_ clusters onto H‐terminated planar p‐Si(100) and p‐Si‐µWs substantially enhanced the PEC performance for HER.^169^ Although no high temperature post‐treatment was applied, the Mo_3_S_4_‐cast p‐Si photocathode still exhibited an operational stability of 24 h, confirming the robust anchoring the Mo_3_S_4_ clusters on Si. Huang et al. first reported the coupling of TMP catalysts to photoelectrodes for PEC water splitting. They prepared a suspension containing Ni_12_P_5_ NPs synthesized hydrothermally,^170^ and simply drop‐cast the solution on SiNW arrays. The NPs were distributed nicely on the sidewall of SiNWs (**Figures** **13**a,b), and no aggregation was found. The as‐fabricated Ni_12_P_5_/SiNWs photocathode showed significantly improved PEC performance in comparison to the bare SiNWs (Figure 13c), achieving a conversion efficiency of 2.97%. A post‐treatment at 450 °C in an inert‐reductive atmosphere enhanced the robustness of Ni_12_P_5_‐SiNWs coupling, and the resulting photocathodes demonstrated good stability in the course of 1 h. Hu Liu et al. synthesized CoSe_2_ NR HER catalysts via hydrothermal process and then spin‐coated these CoSe_2_ NRs onto Si‐µW array photocathodes (Figures d,e). Due likely to the big size, the CoSe_2_ NRs were only found to deposit on the top part of the µW array. Nevertheless, markedly improved PEC performance was still achieved (Figure 13f).^171^ Lewis and co‐workers also managed to load CoP NP and Pt NP catalysts on ordered Si‐µW arrays,^172^ and they found that to achieve similar geometric area‐based PEC performance, the loading of CoP NPs should be significantly higher than that of Pt NPs, given the lower turnover frequency of CoP catalysts. Besides, many other semiconductor/electrocatalyst couples, such as MoS_2_ NSs on TiO_2_ NR arrays,^173^ NiFe LDH on hematite,^174^ MoS_3_ on organic/inorganic CuI/P3HT:PCBM,^175^ cobalt nitride NSs on BiVO_4_,^176^ CoP NPs on hematite,^177^ MoS_2_ on Cu_2_O,^178^ and Ni_2_P on Si,^179^ were also realized by drop casting or spin coating, all of which showed enhanced PEC performance. However, it should be noted that drop casting or spin coating of nanostructured catalysts does not render the formation of a continuous catalyst layer on semiconductor photoelectrodes, and the interfacial contact between semiconductor and electrocatalyst is generally poor. Therefore, it is challenging to achieve long‐term operational stability with this kind of photoelectrode configuration.

In addition to loading active electrocatalysts directly on semiconductor photoelectrodes through droplet‐based methods, the semiconductor/electrocatalyst coupling can also be realized via a two‐step process, namely, drop/spin/spray‐coating precursors on semiconductors, followed by a post‐treatment to convert precursors into active electrocatalysts. For example, by spin coating of methanol solution containing (NH_4_)_2_MoS_4_ powders on SiNW arrays, followed by thermal pyrolysis at 260 °C for 2 h in inert atmosphere, Huang et al. successfully coupled MoS_3_ cluster catalysts to SiNW photocathodes. Thus‐prepared SiNWs@MoS_3_ photocathode exhibited superb performance for PEC H_2_ production with an ABPE of 2.28%, 5700% larger than that of pristine SiNWs (0.039%) and only ≈13% smaller than that of SiNWs loaded with Pt NPs (2.61%).^64^ Using a similar strategy, the same group also loaded WS_2_,^180^ WS_3_,^180^ and FeP catalysts^181^ on SiNW array photocathodes. In all cases, the catalyst formed discrete NPs on the sidewall of SiNWs, efficiently facilitating the PEC H_2_ evolution. Recently, Liu and co‐workers reported the conformal and continuous deposition of CoP catalysts on a spatially ordered SiNW array photocathode.^182^ They found that a simple drop casting of Co‐containing solution and subsequent phosphorization allowed for the formation of conformal coating of nanocrystalline Co_2_P which covered the entire surface of SiNWs (Figure 13g). The as‐deposited Co_2_P layer not only served as a highly active HER catalyst but effectively passivated Si against corrosion, in such a way both the PEC HER activity and stability were remarkably improved (Figure 13h). Furthermore, using a similar method the same group recently demonstrated that a Co_2_P layer with a thickness gradient can be conformally and continuously deposited on inverted pyramid‐textured Si photocathode.^183^ The thickness‐gradient Co_2_P layer enabled partial spatial decoupling of light‐absorption and catalytic activity, and in this way the photocathode could deliver a photocurrent density as high as 35.2 mA cm^−2^ at 0 V_RHE_ and show excellent stability of 150 h at a high photocurrent density of above 30 mA cm^−2^ under PEC operational conditions.

Likewise, metal oxide and hydroxide catalysts, such as CoO*_x_*,^184^, ^185^ NiO*_x_*,^186^ NiOOH,^187^ and NiFeO*_x_*,^188^ can also be coupled to semiconductor photoanodes via two‐step droplet‐based deposition. Ager et al. developed a nitrogen flow–assisted electrostatic spray pyrolysis process to deposit CoO*_x_* and Ni‐doped CoO*_x_* catalyst layers on BiVO_4_ photoanodes.^184^ They designed a coaxial nozzle where the spray solution was pumped into the inner tube and pressurized nitrogen gas was blown through the outer tube. Upon applying an external bias, the nitrogen gas carries the spray solution to the heated substrate, in such a way high surface coverage of catalysts throughout the porous BiVO_4_ electrode can be achieved. Accordingly, the onset potential was shifted negatively by ≈420 mV and the photocurrent density reached 2.01 mA cm^−2^ at 1.23 V_RHE_. Yoo et al. spray‐coated NiCl_2_‐containing precursors on an indium tin oxide (ITO) protected *n*‐Si/SiO*_x_* photoanode, and found that the as‐coated precursor was converted into an amorphous and porous NiOOH layer upon electrochemical activation in alkaline solution. The obtained *n*‐Si/SiO*_x_*/ITO/a‐NiOOH photoanode exhibited a low photocurrent onset potential of ≈0.98 V_RHE_, a high saturation photocurrent density of 36.98 mA cm^−2^, and a photocurrent density of 27.4 mA cm^−2^ at 1.23 V_RHE_.

Light‐induced decomposition is also an approach enabling the conversion of drop‐cast or spin‐coated precursors into active electrocatalysts, other than thermal pyrolysis. Berlinguette et al. developed a photochemical metal‐organic deposition (PMOD) method,^189^ by which a variety of amorphous metal oxide OER catalysts can be obtained by spin coating of metal‐organic precursors on a substrate of interest, followed by irradiation under UV light to decompose the organic ligands and subsequent low‐temperature annealing. The resulting mixed‐metal oxides not only show a low overpotential and Tafel slope for OER in alkaline condition, but also possess the favorable properties like abundant active sites, uniform thickness, and conformal distribution.^189^ Employing PMOD, Wang et al. deposited NiFeO*_x_* as a catalyst on hematite photoanodes.^190^ A high photovoltage of 0.61 V was generated, and the photocurrent onset was decreased dramatically to 0.62 V_RHE_. Such prominent performance was ascribed to the minimization of the surface Fermi level pinning effect. When a SiNW/hematite dual‐absorber was used, a record‐low onset potential of 0.32 V_RHE_ was achieved. PMOD was also recently utilized to couple NiO*_x_* to Mo‐doped BiVO_4_ photoanodes,^191^ where improved PEC performance was demonstrated in neutral electrolyte.

Additionally, drop casting also allows complex precursor solutions to be loaded on semiconductor photoelectrodes generating a multi‐function layer. For instance, Reisner's group reported that molecular single‐source precursors, e.g., [Ti_2_(OEt)_9_(NiCl)_2_] and [Ti_4_O(OEt)_15_(CoCl)], can be drop‐cast on various photoelectrodes including Si, WO_3_, and BiVO_4_.^192^ Upon hydrolysis, the coated precursors will form a composite TiNi or TiCo layer consisting of amorphous TiO_2_ and Ni/Co species. The amorphous TiO_2_ can effectively protect semiconductor from corrosion, while the Ni or Co species are known active HER and OER catalysts. Using this simple procedure, both the PEC activity and photoelectrode stability can be markedly improved.

## 4 Summary and Outlook

Unassisted PEC water splitting holds substantial promise for converting intermittent solar energy into storable and dispensable hydrogen fuel, providing the society a clean, sustainable, and carbon‐neutral energy source. The STH conversion efficiency is one of the most important performance indicators of a PEC device. The Department of Energy (DOE), United States, has set out an ultimate STH efficiency target of 25% for PEC water splitting based on photoelectrode systems with concentrated solar irradiation.^193^ The STH efficiency (η_STH_) is defined as follows^194^

(2) $$\eta_{\text{STH}}=\frac{E_{0}\left(1.23\;\text{V}\right)\times j_{\text{op}}\left(\text{mA}\;{\text{cm}}^{-\text{2}}\right)\times f_{\text{FE}}}{P_{\text{in}}}$$

where *E*_0_ = 1.23 V is the thermodynamic potential for water splitting under standard conditions, *j*_op_ stands for the operating photocurrent density, *f*_FE_ represents the Faradaic efficiency which is usually close to 100%, and *P*_in_ is the power density of total incident solar irradiance. To achieve a high η_STH_, improving *j*_op_ is crucial in which coupling efficient HER and OER electrocatalysts to semiconductor photoelectrodes play a significant role, as evidenced by many previous literature reports. In this article, we comprehensively review the strategies developed by far for coupling electrocatalysts of different kinds including metals, alloys, and compounds, to a variety of semiconductor materials with different structures such as flat wafers and 3D nanostructured electrodes. The advantages and disadvantages of each method used for coupling are summarized below in **Table** **1**.

**Table 1:** Summary of pros and cons of different methods developed for semiconductor/electrocatalysts coupling

Methods used for semiconductor/electrocatalyst coupling	Advantages	Disadvantages
Physical vapor deposition (PVD)	▪ Capable of depositing catalysts that cannot be readily prepared by wet chemical approaches ▪ Independent on semiconductor surface chemistry ▪ A variety of catalytic material targets (including metals, alloys, compounds) are available ▪ Precise control over catalyst layer thickness	▪ Not working for high aspect ratio or 3D‐textured semiconductor photoelectrodes ▪ High vacuum is needed ▪ Expensive apparatus is needed ▪ Low deposition rate
Chemical vapor deposition (CVD)	▪ Catalysts can be coupled to irregular surface of semiconductors ▪ Capable of depositing catalysts conformally on semiconductor surface	▪ Deposition is carried out at high temperatures and the intrinsic properties of semiconductors may change ▪ Vacuum is needed
Atomic layer deposition (ALD)	▪ Capable of depositing catalysts on complicated 3D semiconductor photoelectrodes ▪ Precise control over catalyst layer thickness ▪ Enable uniform deposition of ultrasmall nanoparticulate catalysts, even single atoms	▪ Expensive apparatus is needed ▪ Catalyst precursors are usually expensive ▪ Type of catalytic materials that can be deposited is limited ▪ Low deposition rate
Electrochemical deposition (ECD) or photoassisted electrochemical deposition (PECD)	▪ Inexpensive and scalable ▪ Low temperature process	▪ Semiconductors must be chemically and electrochemically stable in electrolyte ▪ Inadequate control over the catalyst morphology and dispersion
Electroless deposition (ELD)	▪ Simple, inexpensive and scalable ▪ Capable of depositing catalysts on 3D textured semiconductor surface	▪ Process is limited to few semiconductor/catalyst couples ▪ Inadequate control over the catalyst morphology and dispersion
Dip coating	▪ No limitation for semiconductor surface topography ▪ Simple, inexpensive, and scalable ▪ Good control over catalyst layer thickness, allowing for deposition of monolayers	▪ Proper semiconductor surface chemistry is required ▪ Post‐treatment is usually needed to achieve good adhesion ▪ Not applicable for element/alloy catalysts
Successive ionic layer adsorption and reaction (SILAR)	▪ No limitation for semiconductor surface topography ▪ Good control over catalyst layer thickness ▪ Simple, inexpensive, and scalable	▪ Proper semiconductor surface chemistry is required ▪ Suitable for limited number of catalysts
Hydrothermal/Solvothermal treatment	▪ Deposited catalysts have better quality ▪ Nanostructured catalysts can be obtained ▪ No requirement for semiconductor surface chemistry	▪ Not applicable for element/alloy catalysts ▪ Semiconductors must be chemically stable ▪ Inadequate control over catalyst loading
Droplet‐based deposition	▪ No limitation for the type of catalysts to be coupled ▪ No requirement for semiconductor surface chemistry ▪ Simple, inexpensive, and scalable	▪ Inadequate control over the uniformity of catalyst dispersion ▪ Post‐treatment is usually needed to achieve good adhesion

In general, dry processes have been extensively used in semiconductor industry, allowing for precise control of catalyst layer thickness and loading, though they are energy‐demanding and rely on the usage of expensive equipment. Wet chemical processes, by contrast, show much flexibility, and are easy‐to‐implement and significantly less expensive, but the uniformity of catalyst deposition over a large area should be improved.

When choosing appropriate strategies for semiconductor/electrocatalyst coupling, the following considerations need to be taken into account. First, the catalyst coupling should not lead to significant parasitic light absorption. Although for front illumination, parasitic light absorption is inevitable, it must be minimized to a great extent. This requires either the usage of electrocatalysts that are optically transparent (e.g., with a wide band gap or small optical extinction coefficient) or favorable structure designs that allow to minimize the geometric footprints of catalyst or enable spatial decoupling of light absorption and catalytic activity. Second, the coupling should preferably promote, or at least not alter, the interfacial energetics. In this case, methods that do not change the semiconductor intrinsic properties and introduce surface defects should be considered. Third, the processibility should be taken into account according to the nature of electrocatalysts and semiconductors. Some catalysts can only be coupled to semiconductor by a specific method, and some semiconductors may not be chemically or electrochemically stable under the processing conditions of catalyst coupling. Last but not least, the cost‐effectiveness should be considered, in particular for large‐scale fabrication of photoelectrodes, to meet the DOE's electrode cost target.

Notwithstanding many strategies available to accomplish semiconductor/electrocatalyst coupling, new methods that are more reproducible and allow for desirable interfacing between semiconductor and catalysts for enhanced overall PEC water splitting should be continuously developed, to enable PEC water splitting to eventually become a reliable and affordable pathway to solar energy storage.

## Conflict of Interest

The authors declare no conflict of interest.
